# Redundancy in Aromatic *O*-Demethylation and Ring-Opening Reactions in *Novosphingobium aromaticivorans* and Their Impact in the Metabolism of Plant-Derived Phenolics

**DOI:** 10.1128/AEM.02794-20

**Published:** 2021-03-26

**Authors:** Jose M. Perez, Wayne S. Kontur, Carson Gehl, Derek M. Gille, Yanjun Ma, Alyssa V. Niles, German Umana, Timothy J. Donohue, Daniel R. Noguera

**Affiliations:** a Department of Civil and Environmental Engineering, University of Wisconsin–Madison, Madison, Wisconsin, USA; b DOE Great Lakes Bioenergy Research Center, Madison, Wisconsin, USA; c Wisconsin Energy Institute, University of Wisconsin–Madison, Madison, Wisconsin, USA; d Department of Biomedical Engineering, University of Wisconsin–Madison, Madison, Wisconsin, USA; e Department of Bacteriology, University of Wisconsin–Madison, Madison, Wisconsin, USA; Shanghai Jiao Tong University

**Keywords:** *Novosphingobium aromaticivorans*, *O*-demethylases, PDC, aromatic metabolism, biological funneling, dioxygenases, lignocellulose, syringic acid

## Abstract

Lignin is a plant heteropolymer composed of phenolic subunits. Because of its heterogeneity and recalcitrance, the development of efficient methods for its valorization still remains an open challenge. One approach to utilize lignin is its chemical deconstruction into mixtures of monomeric phenolic compounds, followed by biological funneling into a single product. Novosphingobium aromaticivorans DSM 12444 has been previously engineered to produce 2-pyrone-4,6-dicarboxylic acid (PDC) from depolymerized lignin by simultaneously metabolizing multiple aromatics through convergent routes involving the intermediates 3-methoxygallic acid (3-MGA) and protocatechuic acid (PCA). We investigated enzymes predicted to be responsible for *O*-demethylation and oxidative aromatic ring opening, two critical reactions involved in the metabolism of phenolic compounds by *N. aromaticivorans*. The results showed the involvement of DesA in *O*-demethylation of syringic and vanillic acids, LigM in *O-*demethylation of vanillic acid and 3-MGA, and a new *O-*demethylase, DmtS, in the conversion of 3-MGA into gallic acid (GA). In addition, we found that LigAB was the main aromatic ring-opening dioxygenase involved in 3-MGA, PCA, and GA metabolism and that a previously uncharacterized dioxygenase, LigAB2, had high activity with GA. Our results indicate a metabolic route not previously identified in *N. aromaticivorans* that involves *O*-demethylation of 3-MGA to GA. We predict that this pathway channels ∼15% of the carbon flow from syringic acid, with the rest following ring opening of 3-MGA. The new knowledge obtained in this study allowed for the creation of an improved engineered strain for the funneling of aromatic compounds that exhibits stoichiometric conversion of syringic acid into PDC.

**IMPORTANCE** For lignocellulosic biorefineries to effectively contribute to reduction of fossil fuel use, they need to become efficient at producing chemicals from all major components of plant biomass. Making products from lignin will require engineering microorganisms to funnel multiple phenolic compounds to the chemicals of interest, and *N. aromaticivorans* is a promising chassis for this technology. The ability of *N. aromaticivorans* to efficiently and simultaneously degrade many phenolic compounds may be linked to having functionally redundant aromatic degradation pathways and enzymes with broad substrate specificity. A detailed knowledge of aromatic degradation pathways is thus essential to identify genetic engineering targets to maximize product yields. Furthermore, knowledge of enzyme substrate specificity is critical to redirect flow of carbon to desired pathways. This study described an uncharacterized pathway in *N. aromaticivorans* and the enzymes that participate in this pathway, allowing the engineering of an improved strain for production of PDC from lignin.

## Abstract (precis)

For lignocellulosic biorefineries to effectively contribute to reduction of fossil fuel use, they need to become efficient at producing chemicals from all major components of plant biomass. Making products from lignin will require engineering microorganisms to funnel multiple phenolic compounds to the chemicals of interest, and *N. aromaticivorans* is a promising chassis for this technology.

## INTRODUCTION

Lignocellulosic plant biomass, composed of cellulose, hemicellulose, and lignin, is the most abundant organic material on the planet with potential to support a sustainable economy based on renewable feedstocks (1). Numerous studies predict that the economic and environmental viability of lignocellulosic biomass utilization for fuel and chemical production will be increased by the utilization of as much of these polymers as possible, including the use of lignin for production of chemicals (2–4). We are interested in deciphering the bacterial metabolism of phenolic compounds to engineer bacterial hosts to convert biomass-derived lignin into chemicals.

Lignin is an amorphous heteropolymer containing mainly syringyl (S; two methoxy groups), guaiacyl (G; one methoxy group), and *p*-hydroxyphenyl (H; no methoxy groups) phenolic structures that differ in the number of methoxy groups attached to the aromatic ring (5). One approach to valorizing lignin that has gained significant attention is to first use chemical techniques to deconstruct plant biomass and generate mixtures containing a large fraction of lower-molecular-weight phenolic compounds that could then be transformed by engineered microbes to a single valuable product (6). This funneling of phenolic mixtures to single compounds has been demonstrated with engineered strains of Pseudomonas putida (6, 7), Rhodococcus jostii (8), and Novosphingobium aromaticivorans (9). In addition, other microbes, such as the yeast Rhodosporidium toruloides (10) and the photoheterotrophic bacterium Rhodopseudomonas palustris (11–13), have been extensively studied for their ability to transform the plant-derived phenolic compounds often present in deconstructed plant biomass.

Among the desirable features for a microbial strain to be used as a chassis for the development of microbial lignin valorization strategies are an ability to metabolize the majority of the biomass-derived phenolic compounds and to funnel them into native convergent metabolic pathways (14). We are studying the sphingomonad bacterium *N. aromaticivorans* as a platform microbe for lignin valorization because it efficiently and simultaneously utilizes a large variety of S, G, and H phenolics (9, 15) and because it and other sphingomonads have enzymes to cleave different intersubunit bonds in the lignin polymer (16–20). These features, when combined with the genetic and genomic information on sphingomonads like *N. aromaticivorans*, could support strategies to maximize the number and type of lignin depolymerization products (e.g., phenolic monomers, oligomers) that can be microbially transformed into a range of valuable chemicals.

**FIG 1:**
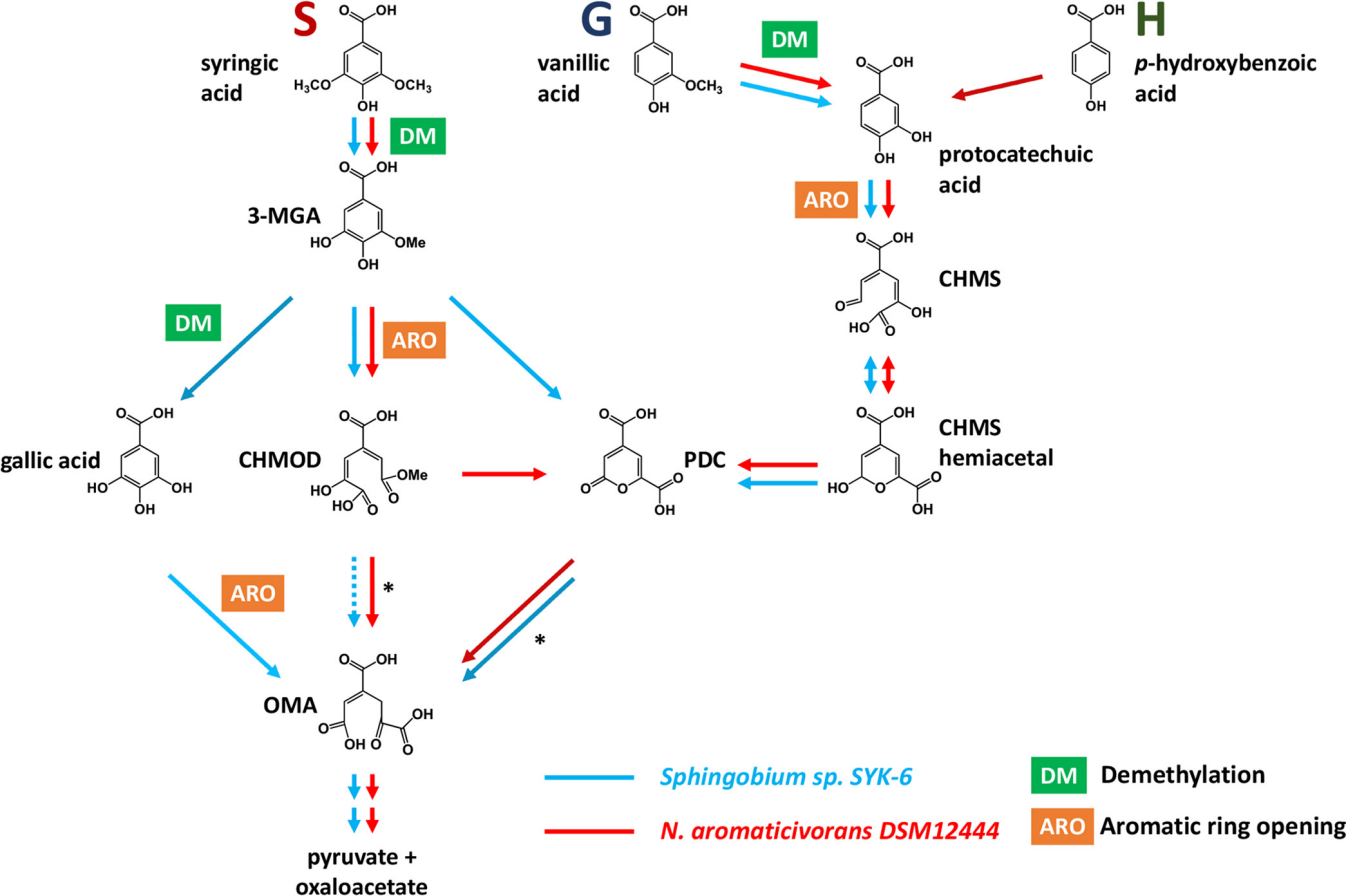
Pathways for the metabolism of S, G, and H phenolics that have been proposed for *Sphingobium* sp. SYK-6 (21–26) and Novosphingobium aromaticivorans (9, 15), showing the location of *O-*demethylation and aromatic ring-opening steps. Abbreviations: 3-MGA, 3-methylgallate; CHMOD, 4-carboxy-2-hydroxy-6-methoxy-6-oxohexa-2,4-dienoate; CHMS, 4-carboxy-2-hydroxy-*cis,cis*-muconate-6-semialdehyde; PDC, 2-pyrone-4,6-dicarboxylic acid; OMA, 4-oxalomesaconate. Asterisks indicate reactions that are disrupted in strain 12444PDC by the deletion of genes *ligI*, *desC*, and *desD* (9).

Metabolic pathways for the degradation of phenolics in sphingomonads have been proposed for *Sphingobium* sp. strain SYK-6, based on experiments with mutant strains and purified enzymes (21–23), and for *N. aromaticivorans*, based on analysis of a genome-scale transposon library and a set of targeted deletion mutants (9, 15). These studies have revealed several commonalities in the phenolic metabolism pathways of both organisms (Fig. 1), but there remain significant knowledge gaps that limit the engineering of strains with increased transformation of phenolics to desired products.

To illustrate some of these knowledge gaps, syringic acid, which is an abundant biomass component that has been analyzed as a model S phenolic in both organisms, is demethylated to 3-methoxygallic acid (3-MGA), after which multiple pathways have been proposed for 3-MGA transformation by *Sphingobium* sp. SYK-6 (21, 22). In one proposed pathway, the demethylation of 3-MGA produces gallic acid (GA), whose aromatic ring is cleaved by a dioxygenase to produce 4-oxalomesaconate (OMA) (24). Alternatively, it is proposed that the aromatic ring of 3-MGA is cleaved by a dioxygenase to produce 4-carboxy-2-hydroxy-6-methoxy-6-oxohexa-2,4-dienoate (CHMOD), which could be converted to OMA by an unknown enzyme (21, 22). Furthermore, *in vitro* experiments with a purified dioxygenase from *Sphingobium* sp. SYK-6 (LigAB) and 3-MGA as the substrate showed rapid production of 2-pyrone-4,6-dicarboxylic acid (PDC) in addition to the ring cleavage product CHMOD, a result that led to the hypothesis that LigAB catalyzes the transformation of 3-MGA to both CHMOD and PDC (22). In *N. aromaticivorans*, the only pathway thus far proposed for syringic acid metabolism (15) is via demethylation to 3-MGA, ring cleavage to CHMOD, and conversion to OMA. However, an *N. aromaticivorans* deletion mutant lacking the proposed enzymes for CHMOD conversion to OMA (DesC and DesD) resulted in accumulation of PDC, suggesting the possibility for cyclization of CHMOD to PDC that is independent of these enzymes (9).

There are also knowledge gaps in the bacterial metabolism of the other major G and H phenolic substituents of plant cell walls. In the case of the G phenolic vanillic acid, its metabolism by both *Sphingobium* sp. SYK-6 and *N. aromaticivorans* is proposed (15, 16) to entail demethylation to protocatechuic acid (PCA), ring cleavage to 4-carboxy-2-hydroxy-*cis*,*cis*-muconate-6-semialdehyde (CHMS), oxidation to PDC, and hydrolysis to OMA (Fig. 1). Degradation of the H phenolic *p*-hydroxybenzoic acid has been studied only in *N. aromaticivorans* (9), with experimental evidence suggesting transformation to PCA as the initial step to enter the described pathway for G phenolics (Fig. 1). However, it is unclear if the S and G/H branches of the phenolic degradation pathways in sphingomonads have common or pathway-specific *O-*demethylation and aromatic ring cleavage enzymes (Fig. 1). Indeed, it has been proposed that some of these demethylase enzymes have activity on more than one substrate and are active in multiple branches. For example, one dioxygenase (LigAB) in *N. aromaticivorans* has been proposed to be active in the ring cleavage of 3-MGA and PCA (15), and in *Sphingobium* sp. SYK-6, an *O-*demethylase (LigM) has been proposed to be active on both 3-MGA and vanillic acid (25).

To address knowledge gaps in the reactions and enzymes involved in S, G, and H phenolic metabolism by *N. aromaticivorans*, we analyzed putative *O-*demethylases and aromatic ring opening dioxygenases that are predicted to be involved in the metabolism of these plant-derived aromatics. We present results of experiments with purified enzymes and with deletion mutants that provide evidence for functionally redundant pathways and for enzymes with activity on more than one substrate in this organism. Our studies led us to identify an aromatic ring-opening dioxygenase (LigAB2) and an *O-*demethylase (DmtS) that have not been previously shown to have these activities in sphingomonads. In addition, the newly acquired knowledge on enzyme redundancy and substrate specificity allowed us to engineer a second-generation *N. aromaticivorans* DSM 12444 strain with improved yields of PDC from plant-derived phenolics.

## RESULTS

### Identification of putative aromatic *O-*demethylases in *N. aromaticivorans*.

We evaluated gene products with significant amino acid sequence identity to *O-*demethylases, encoded by Saro_2861 (*ligM*) and Saro_2404 (*desA*), that have been proposed to be involved in vanillic and syringic acid metabolism, respectively (15). These two proteins share ∼78% and ∼71% amino acid sequence identity with the *Sphingobium* sp. SYK-6 *O-*demethylases SLG_12740 (*ligM*) (25) and SLG_25000 (*desA*) (26). In addition, since *O-*demethylation of vanillic acid in *Pseudomonas* (27, 28) is catalyzed by VanAB, we analyzed the *N. aromaticivorans* gene Saro_1872 (hereafter called *dmtS*), which encodes a protein with the closest amino acid sequence identity to the VanA subunit of this enzyme (e.g., ∼27% amino acid identity with VanA of *Pseudomonas* sp. strain HR199 [29]).

### (i) Effect of deleting putative *O*-demethylase genes on *N. aromaticivorans* growth.

To begin evaluating the involvement of *ligM*, *desA*, and *dmtS* in the degradation of S and G phenolics in *N. aromaticivorans*, we generated mutants (Table 1) containing combinations of deletions of these three genes in a parent strain (12444 Δ*1879*) and in a strain (12444PDC) in which deletions in PDC and CHMOD degradation genes led to accumulation of PDC from S, G, and H aromatics (9).

**TABLE 1:** *N. aromaticivorans* parent and mutant strains with deletions of putative *O-*demethylases used in this study

Strain	Background used for strain construction	Relevant characteristics
Parent strain (12444 Δ*1879*)^*a*^	DSM 12444	12444 Δ*1879*
PDC-producing strain (12444PDC)^*a*^	12444 Δ*1879*	12444 Δ*1879* Δ*Saro_2819* Δ*Saro_2864* Δ*Saro_2865*
12444 Δ*ligM*	12444 Δ*1879*	12444 Δ*1879* Δ*Saro_2861*
12444 Δ*desA*	12444 Δ*1879*	12444 Δ*1879* Δ*Saro_2404*
12444 Δ*dmtS*	12444 Δ*1879*	12444 Δ*1879* Δ*Saro_1872*
12444 Δ*ligM* Δ*desA*	12444 Δ*ligM*	12444 Δ*1879* Δ*Saro_2861* Δ*Saro_2404*
12444 Δ*ligM* Δ*dmtS*	12444 Δ*ligM*	12444 Δ*1879* Δ*Saro_2861* Δ*Saro_1872*
12444 Δ*desA* Δ*dmtS*	12444 Δ*dmtS*	12444 Δ*1879* Δ*Saro_2404* Δ*Saro_1872*
12444 Δ*ligM* Δ*desA* Δ*dmtS*	12444 Δ*ligM* Δ*dmtS*	12444 Δ*1879* Δ*Saro_2861* Δ*Saro_2404* Δ*Saro_1872*
12444PDC Δ*ligM*	12444PDC	12444PDC Δ*Saro_2861*
12444PDC Δ*desA*	12444PDC	12444PDC Δ*Saro_2404*
12444PDC Δ*dmtS*	12444PDC	12444PDC Δ*Saro_1872*
12444PDC Δ*ligM* Δ*desA*	12444PDC Δ*ligM*	12444PDC Δ*Saro_2861* Δ*Saro_2404*
12444PDC Δ*ligM* Δ*dmtS*	12444PDC Δ*ligM*	12444PDC Δ*Saro_2861* Δ*Saro_1872*
12444PDC Δ*desA* Δ*dmtS*	12444PDC Δ*dmtS*	12444PDC Δ*Saro_2404* Δ*Saro_1872*
12444PDC Δ*ligM* Δ*desA* Δ*dmtS*	12444PDC Δ*ligM* Δ*dmtS*	12444PDC Δ*Saro_2861* Δ*Saro_2404* Δ*Saro_1872*

a Construction of the parent and PDC-producing strains was described previously by Perez et al. (9). The parent strain has a deletion of Saro_1879 (*sacB*) to render it susceptible to genetic manipulation using *sacB* as a counterselection marker (9).

**FIG 2:**
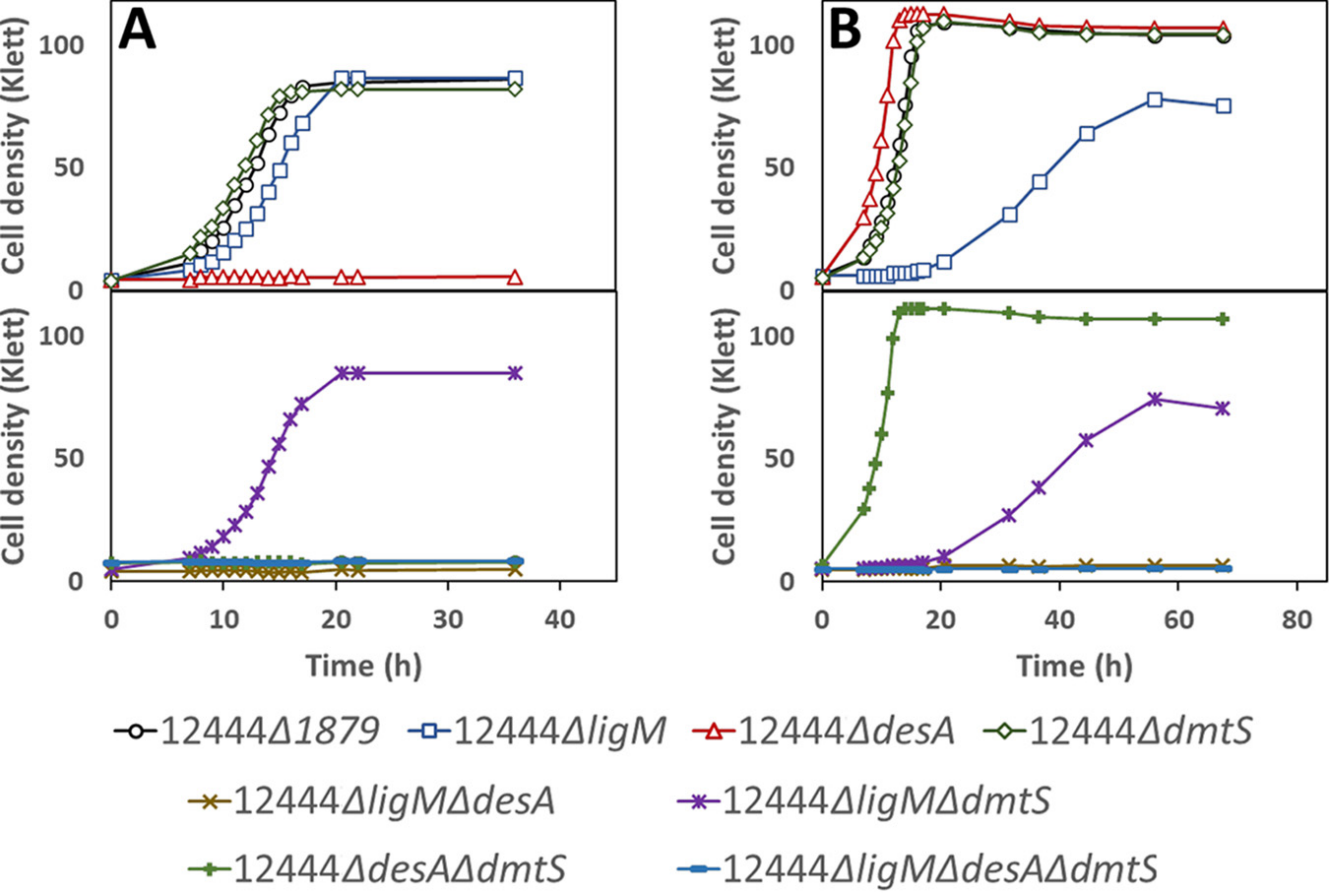
Growth of indicated strains of *N. aromaticivorans* in minimal medium supplemented with 3 mM syringic acid (A) or 3 mM vanillic acid (B). Top panels show data for parent strain (12444 Δ*1879*) and single deletion mutants, whereas bottom panels show growth curves for strains with multiple gene deletions. All strains contain the Δ*1879* gene deletion. See Table 1 for the definition of strain names. Values correspond to the average results for three biological replicates.

Figure 2 shows growth curves for the parent strain and for its corresponding set of mutant strains. When cultured in the presence of only syringic acid, the parent strain and the mutant strains 12444 Δ*ligM*, 12444 Δ*dmtS*, and 12444 Δ*ligM* Δ*dmtS* had similar growth patterns, whereas all mutant strains lacking *desA* were unable to grow (Fig. 2A). This suggests that *desA* is essential for *N. aromaticivorans* growth on syringic acid, whereas *ligM* and *dmtS* are not.

When cultured in the presence of vanillic acid as the sole carbon source, three distinct growth patterns were observed for this set of mutants (Fig. 2B). First, all mutant strains with an intact *ligM* (12444 Δ*desA*, 12444 Δ*dmtS*, and 12444 Δ*desA* Δ*dmtS*) exhibited a similar growth pattern as the parent strain, suggesting that the DesA and DmtS enzymes are not essential for *N. aromaticivorans* growth on vanillic acid. Second, strains 12444 Δ*ligM* and 12444 Δ*ligM* Δ*dmtS* showed lower growth rates and a lower final cell density than the parent strain, and third, strains simultaneously lacking *desA* and *ligM*, 12444 Δ*desA* Δ*ligM* and 12444 Δ*desA* Δ*ligM* Δ*dmtS*, were unable to grow in the presence of vanillic acid.

Because the growth defects caused by gene deletions in the parent strain do not necessarily reveal which specific intracellular reactions are being affected, and can obscure the roles of genes with redundant functions, we also used the PDC-producing strain as a background to construct mutants lacking the same combinations of putative *O-*demethylase genes (Table 1). With this second set of mutants, which require a nonaromatic carbon source for growth, we used PDC production as a proxy to elucidate the metabolic pathways affected by the gene deletions.

**FIG 3:**
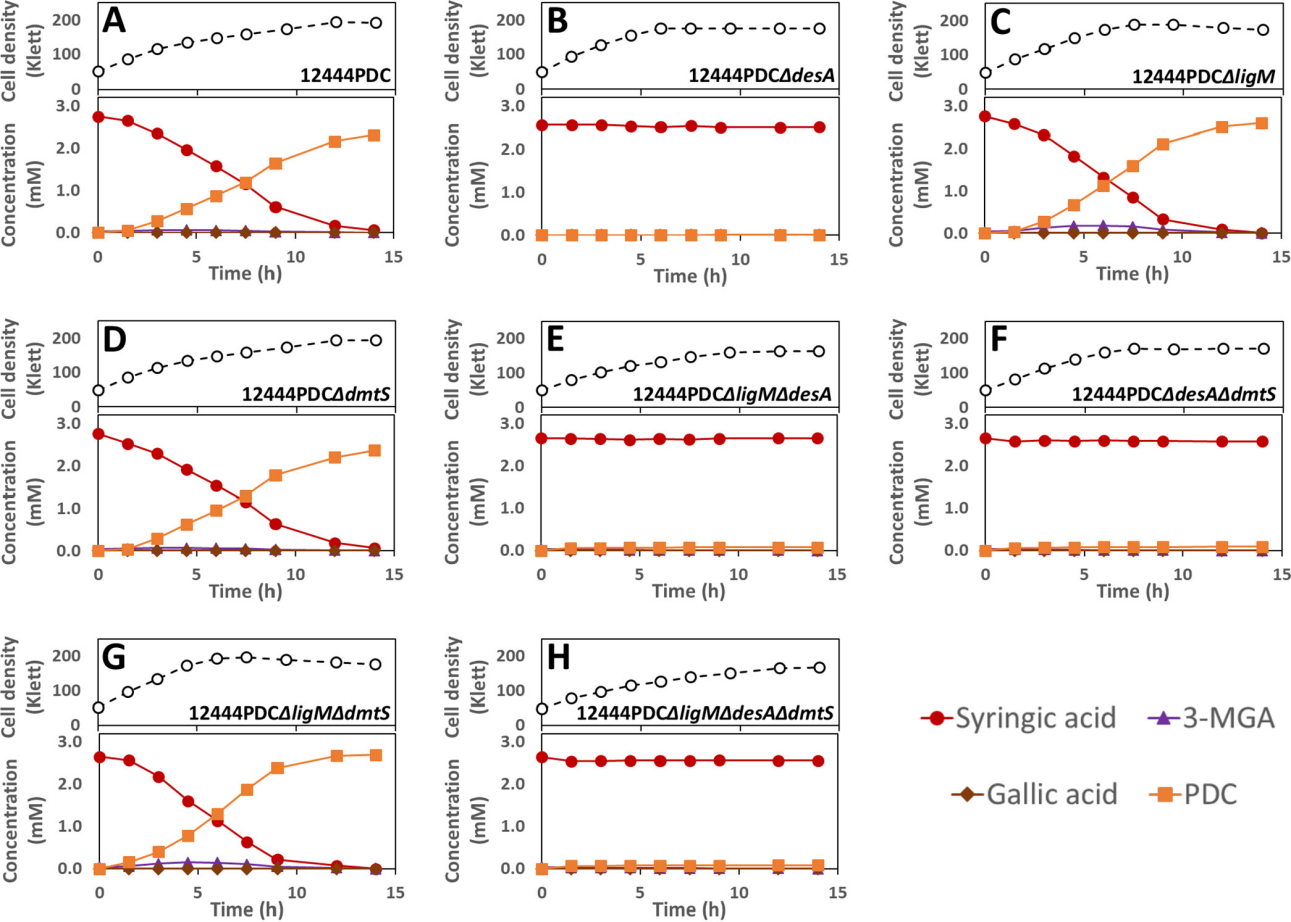
Cell densities and extracellular compound concentrations for *N. aromaticivorans* strains 12444PDC (A), 12444PDC Δ*desA* (B), 12444PDC Δ*ligM* (C), 12444PDC Δ*dmtS* (D), 12444PDC Δ*ligM* Δ*desA* (E), 12444PDC Δ*desA* Δ*dmtS* (F), 12444PDC Δ*ligM* Δ*dmtS* (G), and 12444PDC Δ*ligM* Δ*desA* Δ*dmtS* (H), grown in minimal medium containing 3.2 mM glucose and 3 mM syringic acid. All strains contain the Δ*1879* gene deletion. See Table 1 for the definition of strain names. Values correspond to the average results for three biological replicates. Error bars are included but in most cases not visible because they are smaller than the symbols in the plots. Abbreviations: 3-MGA, 3-methylgallate; PDC, 2-pyrone-4,6-dicarboxylic acid.

In the presence of glucose and syringic acid as an aromatic carbon source, the original PDC-producing strain (12444PDC) completely removed the syringic acid from the medium and produced PDC with a yield of ∼85% (Fig. 3A; Table 2). All strains with deletion of *desA* (12444PDC Δ*desA*, 12444PDC Δ*desA* Δ*ligM*, 12444PDC Δ*desA* Δ*dmtS*, and 12444PDC Δ*desA* Δ*ligM* Δ*dmtS*) were not able to degrade syringic acid (Fig. 3B, E, F and H). These results support an essential role for DesA in syringic acid metabolism by *N. aromaticivorans*, which is proposed to be its demethylation to 3-MGA (Fig. 1).

**TABLE 2:** PDC yields from *N. aromaticivorans* strains in the presence of glucose plus the indicated aromatic substrate^*a*^

Aromatic substrate	Strain^*b*^	Deleted gene(s)			PDC yield (%)^*c*^
		Saro_2404 (*desA*)	Saro_2861 (*ligM*)	Saro_1872 (*dmtS*)	
Syringic acid	12444PDC				84.8 ± 0.6
	12444PDC Δ*ligM*		×		93.0 ± 0.8
	12444PDC Δ*desA*	×			N/C^*d*^
	12444PDC Δ*dmtS*			×	86.5 ± 1.5
	12444PDC Δ*ligM* Δ*desA*	×	×		N/C
	12444PDC Δ*ligM* Δ*dmtS*		×	×	100.4 ± 2.5
	12444PDC Δ*desA* Δ*dmtS*	×		×	N/C
	12444PDC Δ*ligM* Δ*desA* Δ*dmtS*	×	×	×	N/C
3-MGA	12444PDC				93.7 ± 0.3
	12444PDC Δ*ligM*		×		97.2 ± 0.3
	12444PDC Δ*desA*	×			93.8 ± 0.4
	12444PDC Δ*dmtS*			×	94.0 ± 0.1
	12444PDC Δ*ligM* Δ*desA*	×	×		98.1 ± 1.0
	12444PDC Δ*ligM* Δ*dmtS*		×	×	100.1 ± 0.8
	12444PDC Δ*desA* Δ*dmtS*	×		×	90.9 ± 0.7
	12444PDC Δ*ligM* Δ*desA* Δ*dmtS*	×	×	×	100.7 ± 0.7
Vanillic acid	12444PDC				95.9 ± 1.5
	12444PDC Δ*ligM*		×		92.8 ± 2.6
	12444PDC Δ*desA*	×			98.0 ± 3.0
	12444PDC Δ*dmtS*			×	92.8 ± 1.8
	12444PDC Δ*ligM* Δ*desA*	×	×		47.1 ± 6.7
	12444PDC Δ*ligM* Δ*dmtS*		×	×	97.8 ± 3.0
	12444PDC Δ*desA* Δ*dmtS*	×		×	99.4 ± 1.3
	12444PDC Δ*ligM* Δ*desA* Δ*dmtS*	×	×	×	34.5 ± 4.3

a All experiments used 3.2 mM glucose and a 3 mM concentration of the corresponding aromatic substrate as organic carbon sources.

b Strains are defined in Table 1.

c Yield reported as average ± standard deviation of results for three biological replicates.

d N/C, PDC yield was not calculated for conditions under which the aromatic substrate was not consumed.

Furthermore, all the mutant strains with intact *desA* that were constructed in the PDC-producing background (12444PDC Δ*ligM*, 12444PDC Δ*dmtS*, and 12444PDC Δ*ligM* Δ*dmtS*) consumed syringic acid and accumulated PDC. Notably, the PDC yield from syringic acid by strain 12444PDC Δ*ligM* Δ*dmtS* was stoichiometric and in all cases statistically higher (*P* < 0.05) (see Table S1 in the supplemental material) than the yield of this product obtained in the other strains (Table 2). This suggests that the simultaneous deletion of both *ligM* and *dmtS* blocks another previously unknown pathway for syringic acid metabolism that normally detracts from PDC production in the 12444PDC strain.

**FIG 4:**
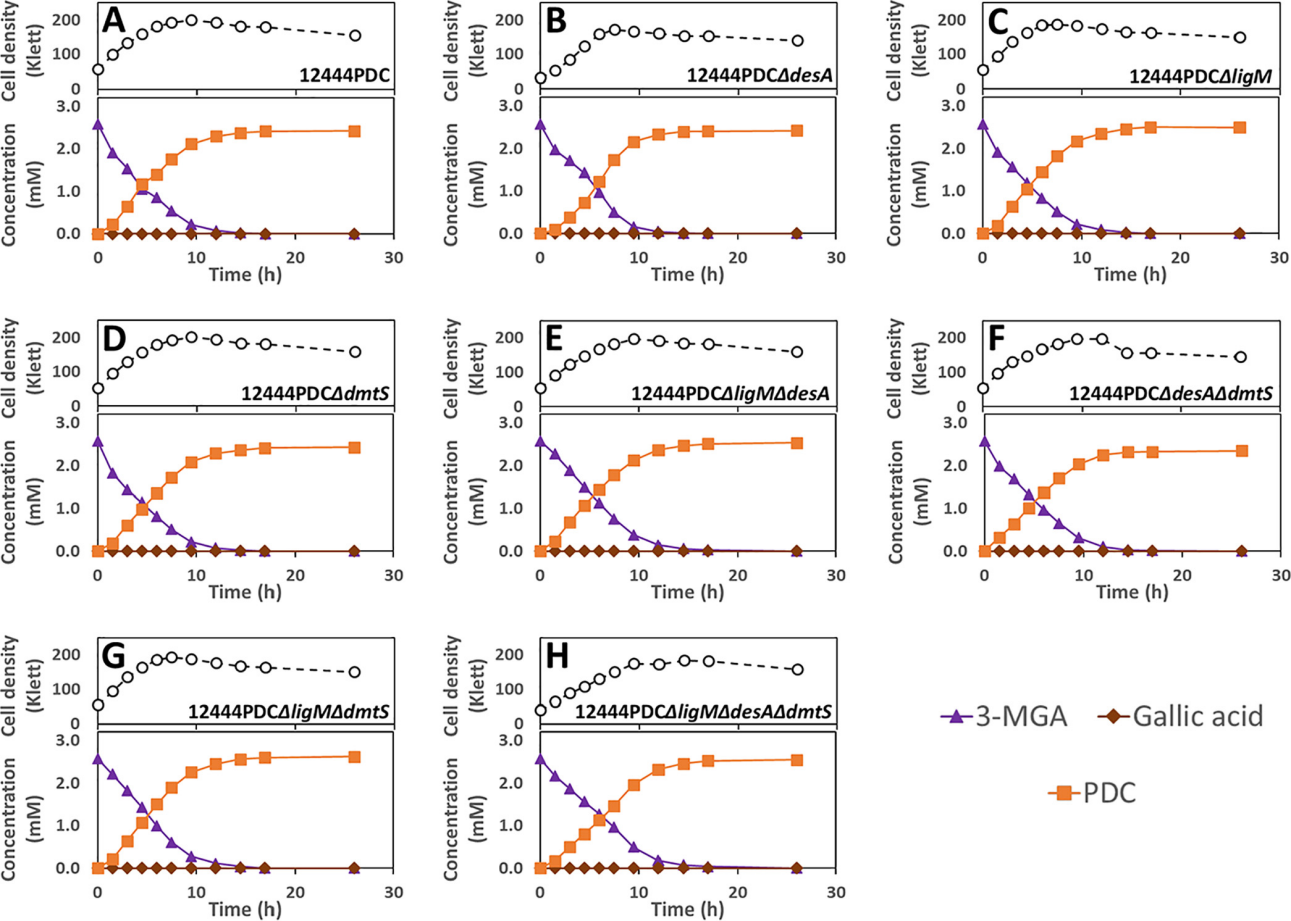
Growth and extracellular aromatic compound concentrations for *N. aromaticivorans* strains 12444PDC (A), 12444PDC Δ*desA* (B), 12444PDC Δ*ligM* (C), 12444PDC Δ*dmtS* (D), 12444PDC Δ*ligM* Δ*desA* (E), 12444PDC Δ*desA* Δ*dmtS* (F), 12444PDC Δ*ligM* Δ*dmtS* (G), and 12444PDC Δ*ligM* Δ*desA* Δ*dmtS* (H), grown in minimal medium supplemented with 3.2 mM glucose and 3 mM 3-MGA. All strains contain the Δ*1879* gene deletion. See Table 1 for the definition of strain names. Values correspond to the average results for three biological replicates. Error bars are included but in most cases not visible because they are smaller than the symbols in the plots. Abbreviations: 3-MGA, 3-methylgallate; PDC, 2-pyrone-4,6-dicarboxylic acid.

We predict that this alternative pathway for syringic acid metabolism involves *O-*demethylation (by LigM and/or DmtS) of 3-MGA to GA, since that transformation has been shown to occur in *Sphingobium* sp. SYK-6 (Fig. 1) (25). An observed small and transient accumulation of 3-MGA in the culture medium when 12444PDC Δ*ligM* and 12444PDC Δ*ligM* Δ*dmtS* (Fig. 3C and G) are grown in the presence of syringic acid supports this hypothesis. To further test this hypothesis, we grew the 12444PDC strain and its corresponding set of putative *O-*demethylase deletion mutants in the presence of glucose and 3-MGA (Fig. 4). We posited that using 3-MGA as the aromatic substrate would provide a growth substrate that requires only one demethylation reaction, compared to growth with syringic acid that is predicted to require the removal of two methyl groups. In this set of experiments, strain 12444PDC was able to completely remove 3-MGA from the medium, converting ∼94% of it into PDC. Moreover, in contrast with the experiments using syringic acid (Fig. 3), each of the putative *O-*demethylase mutant derivatives of 12444PDC were able to completely remove 3-MGA from the medium (Fig. 4). This result indicates that none of these putative *O-*demethylases is essential for conversion of 3-MGA to PDC. In addition, all strains with deletions of *ligM* (12444PDC Δ*ligM*, 12444PDC Δ*desA* Δ*ligM*, 12444PDC Δ*ligM* Δ*dmtS*, and 12444PDC Δ*desA* Δ*ligM* Δ*dmtS*) had statistically higher PDC production (*P* < 0.05) (Table S1) than strain 12444PDC (Table 2), a result that suggests the involvement of LigM in the proposed alternative pathway of syringic acid metabolism. Notably, stoichiometric conversion of 3-MGA to PDC was achieved in the mutants with simultaneous deletion of *ligM* and *dmtS* (12444PDC Δ*ligM* Δ*dmtS* and 12444PDC Δ*desA* Δ*ligM* Δ*dmtS*), which, in agreement with the experiments using syringic acid, suggests that the loss of LigM and DmtS blocks the alternative pathway for syringic acid metabolism in this bacterium.

**FIG 5:**
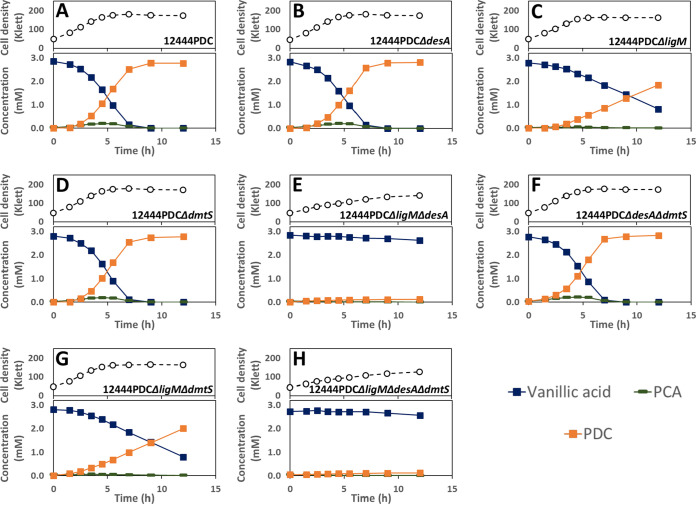
Growth and extracellular aromatic compound concentrations for *N. aromaticivorans* strains 12444PDC (A), 12444PDC Δ*desA* (B), 12444PDC Δ*ligM* (C), 12444PDC Δ*dmtS* (D), 12444PDC Δ*ligM* Δ*desA* (E), 12444PDC Δ*desA* Δ*dmtS* (F), 12444PDC Δ*ligM* Δ*dmtS* (G), and 12444PDC Δ*ligM* Δ*desA* Δ*dmtS* (H), grown in minimal medium supplemented with 3.2 mM glucose and 3 mM vanillic acid. All strains contain the Δ*1879* gene deletion. See Table 1 for the definition of strain names. Values correspond to the average results for three biological replicates. Error bars are included but in most cases not visible because they are smaller than the symbols in the plots. Abbreviations: PCA, protocatechuic acid; PDC, 2-pyrone-4,6-dicarboxylic acid.

In an additional experiment with the PDC-producing strain and its derivatives, we analyzed the role of the putative *O-*demethylases in the metabolism of vanillic acid. In the presence of glucose and vanillic acid, strain 12444PDC completely removed the vanillic acid from the medium and converted it into PDC with ∼96% yield (Table 2). Transient extracellular accumulation of a small amount of PCA during the course of the experiment (Fig. 5A) supports the predicted role of demethylation in the degradation of vanillic acid (Fig. 1). Analysis of the mutants lacking single putative *O-*demethylases (Fig. 5B to D) revealed a decrease in the vanillic acid consumption rate when *ligM* was deleted (Fig. 5C), compared to the other two strains, suggesting a role for LigM in vanillic acid metabolism. In the set of double *O-*demethylase mutants (Fig. 5E to G), no effect was seen when *desA* and *dmtS* were deleted (Fig. 5F), but a decrease in vanillic acid consumption rates was evident with all double mutants that lacked the *ligM* gene. This observation is also consistent with the reduced rate of vanillic acid degradation in the single *ligM* deletion mutant (Fig. 5C). Notably, the rate of vanillic acid consumption decreased the most when *ligM* and *desA* were both deleted (Fig. 5E), suggesting that DesA can partially substitute for LigM in the degradation of vanillic acid. Finally, minimal vanillic acid degradation was observed in a mutant that lacked all three of the putative *O-*demethylase genes (Fig. 5H), consistent with the lack of LigM and DesA activity in this strain.

### (ii) Activity of LigM and DesA with aromatic substrates.

*In vitro* experiments were performed with purified recombinant LigM and DesA proteins to test their activity with the methoxylated aromatic substrates. These assays were performed only with LigM and DesA, since we have thus far been unsuccessful in purifying an active recombinant DmtS protein. The LigM and DesA homologues from *Sphingobium* sp. SYK-6 have been shown to be tetrahydrofolate (H_4_folate)-dependent *O-*demethylases (25, 26), a prediction that was experimentally confirmed (Fig. S1).

**FIG 6:**
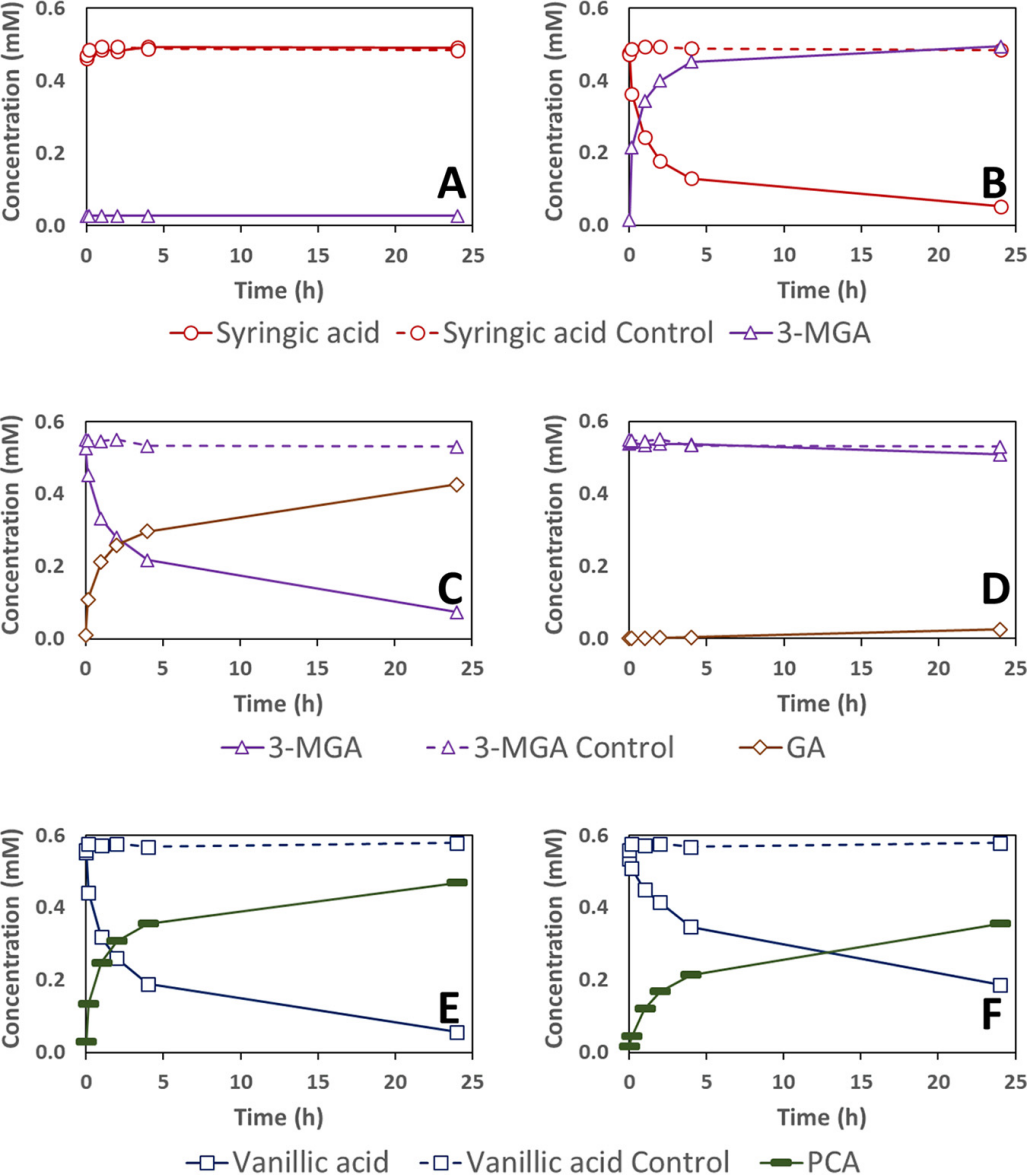
Substrate and product concentration during *in vitro* enzyme assays of LigM (Saro_2861) (A, C, and E) and DesA (Saro_2404) (B, D, and F) with syringic acid (A and B), 3-MGA (C and D), and vanillic acid (E and F). Concentrations in reaction mixtures lacking recombinant proteins are shown by a dashed line. Reported concentrations are the average results of three replicate assays. The low amount of 3-MGA measured in the assays with LigM and syringic acid in panel A was a trace contaminant in the syringic acid used in these assays. Abbreviation: 3-MGA, 3-methylgallate; PCA, protocatechuic acid.

The recombinant LigM protein of *N. aromaticivorans* was able to convert 3-MGA into GA and vanillic acid into PCA at comparable rates under our assay conditions (Fig. 6C and E). However, under identical conditions, the recombinant LigM protein was unable to convert a detectable amount of syringic acid into 3-MGA (Fig. 6A). These results are consistent with the observations in growth experiments with mutant strains, which predicted LigM’s involvement in vanillic acid and syringic acid metabolism.

We also found that the recombinant DesA of *N. aromaticivorans* demethylated both syringic acid and, somewhat more slowly, vanillic acid (Fig. 6B and F) but was not active in demethylating 3-MGA (Fig. 6D). These results support the critical role of DesA in the demethylation of syringic acid that was predicted by analyzing growth of mutant strains (Fig. 2) and its potential role in vanillic acid transformation when *ligM* is deleted (Fig. 5) and offer new evidence supporting the hypothesis that this enzyme does not participate in 3-MGA demethylation.

### Identification of putative aromatic ring-opening dioxygenases in *N. aromaticivorans*.

The opening of the aromatic ring is an essential step in the assimilation of plant-derived phenolic compounds into intermediary metabolism (Fig. 1). In *N. aromaticivorans*, the only aromatic ring-opening dioxygenase that has been previously identified is a LigAB homologue, encoded by Saro_2813 (*ligA*) and Saro_2812 (*ligB*) (15), whose subunits have ∼67% and ∼70% amino acid sequence identity with LigA and LigB of *Sphingobium* sp. SYK-6, respectively. A search of the *N. aromaticivorans* genome reveals genes that could encode another aromatic ring-opening dioxygenase encoded by Saro_1233 and Saro_1234 (hereafter referred to as *ligA2* and *ligB2*, respectively), whose subunits have amino acid sequences that are ∼33% and ∼42% identical to the LigA and LigB of *N. aromaticivorans* (41% and 43% amino acid identity to the LigA and LigB of *Sphingobium* sp. SYK-6, respectively).

### (i) Effect of deleting genes encoding putative aromatic ring-opening dioxygenases on growth of *N. aromaticivorans*.

**FIG 7:**
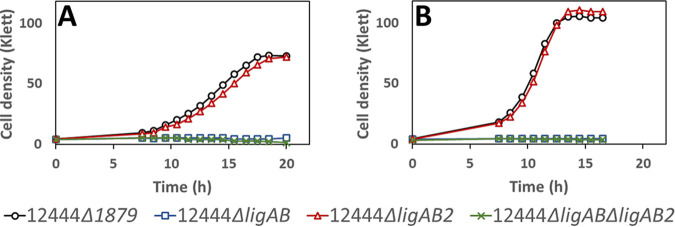
Growth of indicated *N. aromaticivorans* strains in minimal medium supplemented with 3 mM syringic acid (A) or 3 mM vanillic acid (B). All strains contain the Δ*1879* gene deletion. See Table 3 for the definition of strain names. Values correspond to the average results for three biological replicates. Error bars are included but in most cases not visible because they are smaller than the symbols in the plots.

To evaluate the roles of LigAB and LigAB2 in the degradation of S and G phenolics by *N. aromaticivorans*, we tested growth and aromatic metabolism by mutants containing combinations of deletions in *ligAB* and *ligAB2* in the parent strain (12444 Δ*1879*) and the PDC-producing strain (12444PDC) (Table 3). When cultured in the presence of either syringic acid or vanillic acid, the parent strain and strain 12444 Δ*ligAB2* both grew well, whereas strains with deletion of *ligAB* (12444 Δ*ligAB* and 12444 Δ*ligAB* Δ*ligAB2*) were unable to grow (Fig. 7). This indicates that LigAB is necessary for *N. aromaticivorans* growth on both syringic and vanillic acids, whereas LigAB2 is not.

**TABLE 3:** *N. aromaticivorans* mutant strains with deletions of putative aromatic ring cleavage dioxygenases used in this study

Strain	Background used for strain construction	Relevant characteristics
12444 Δ*ligAB*	12444 Δ*1879*^*a*^	12444 Δ*1879* Δ*Saro_2812* Δ*Saro_2813*
12444 Δ*ligAB2*	12444 Δ*1879*	12444 Δ*1879* Δ*Saro_1233* Δ*Saro_1234*
12444 Δ*ligAB* Δ*ligAB2*	12444 Δ*ligAB*	12444 Δ*1879* Δ*Saro_2812* Δ*Saro_2813* Δ*Saro_1233* Δ*Saro_1234*
12444PDC Δ*ligAB*	12444PDC^*a*^	12444PDC Δ*Saro_2812* Δ*Saro_2813*
12444PDC Δ*ligAB2*	12444PDC	12444PDC Δ*Saro_1233* Δ*Saro_1234*
12444PDC Δ*ligAB* Δ*ligAB2*	12444PDC Δ*ligAB*	12444PDC Δ*Saro_2812* Δ*Saro_2813* Δ*Saro_1233* Δ*Saro_1234*

a Construction of the parent strain (12444 Δ*1879*) and the PDC-producing strain (12444PDC) was described previously by Perez et al. (9). See also footnote *a* in Table 1.

**FIG 8:**
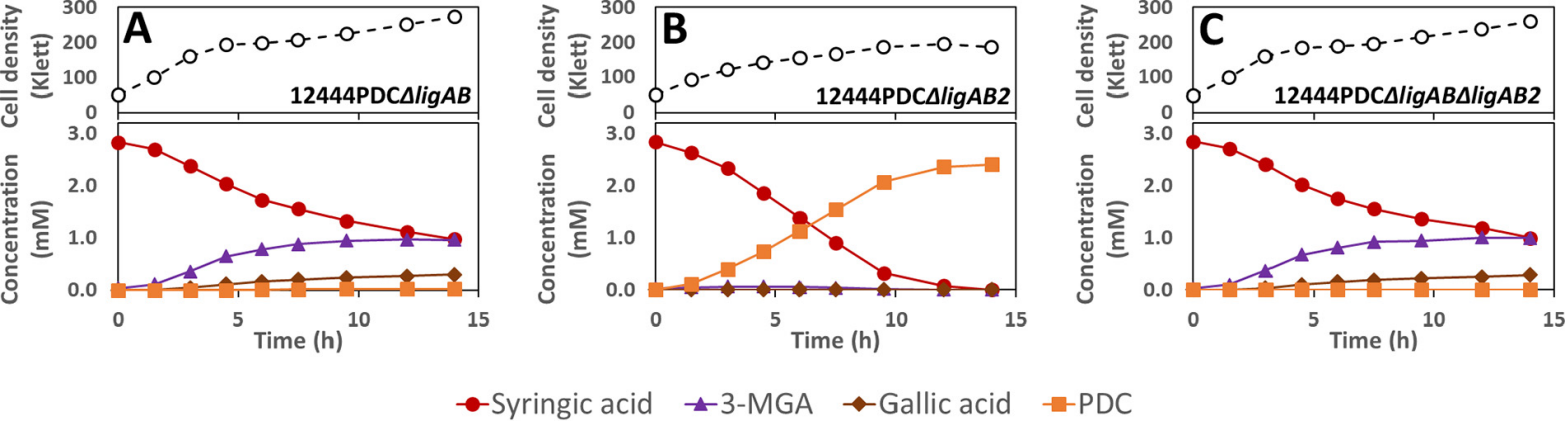
Growth and extracellular compound concentrations for *N. aromaticivorans* strains 12444PDC Δ*ligAB* (A), 12444PDC Δ*ligAB2* (B), and 12444PDC Δ*ligAB* Δ*ligAB2* (C) cultured in minimal medium supplemented with 3.2 mM glucose and 3 mM syringic acid. All strains contain the Δ*1879* gene deletion. See Table 3 for the definition of strain names. Values correspond to the average results for three biological replicates. Error bars are included but in most cases not visible because they are smaller than the symbols in the plots. Abbreviations: 3-MGA, 3-methylgallate; PDC, 2-pyrone-4,6-dicarboxylic acid.

Investigating the metabolism of syringic acid by mutants containing deletions in *ligAB* and *ligAB2* in the PDC-producing strain (12444PDC) (Fig. 8), we observed that all strains consumed syringic acid, but only the mutant with intact LigAB (Fig. 8B) produced PDC with a high yield (∼84%) (Table 4), supporting the hypothesis that LigAB plays an important role in PDC production. The proposed pathway for PDC production from syringic acid includes ring cleavage of 3-MGA to CHMOD, which is then converted to PDC (Fig. 1). The high PDC yield observed in cells in which LigAB is present (Table 4) indicates that this enzyme plays a major role in the transformation of 3-MGA. It also suggests that in *N. aromaticivorans*, 3-MGA ring opening is the primary route for 3-MGA metabolism, while demethylation of 3-MGA to GA is a secondary route responsible for only a small fraction of the metabolized 3-MGA. In support of this, the tests with the two mutants that were missing LigAB (Fig. 8A and C) showed lower rates of syringic acid consumption and accumulation of 3-MGA and GA, the predicted metabolic intermediates in this secondary metabolic route. Strain 12444PDC Δ*ligAB* also converted only ∼2% of the syringic acid to PDC (Table 4), suggesting that LigAB2 may have some role in 3-MGA metabolism, whereas strain12444PDC Δ*ligAB* Δ*ligAB2* did not produce any detectable PDC (Table 4), suggesting a complete interruption of 3-MGA aromatic ring opening in the mutant that lacked both of the putative dioxygenases, LigAB and LigAB2.

**TABLE 4:** PDC yields from *N. aromaticivorans* strains in the presence of glucose plus the indicated aromatic substrate^*a*^

Aromatic substrate	Strain^*b*^	Deleted gene(s)		
		Saro_2812/3 (*ligAB*)	Saro_1233/4 (*ligAB2*)	PDC yield (%)^*c*^
Syringic acid	12444PDC^*d*^			84.8 ± 0.6
	12444PDC Δ*ligAB*	×		2.4 ± 0.6
	12444PDC Δ*ligAB2*		×	84.0 ± 2.1
	12444PDC Δ*ligAB* Δ*ligAB2*	×	×	0.0 ± 0.0
Vanillic acid	12444PDC^*d*^			95.9 ± 1.5
	12444PDC Δ*ligAB*	×		11.6 ± 1.4
	12444PDC Δ*ligAB2*		×	93.3 ± 2.8
	12444PDC Δ*ligAB* Δ*ligAB2*	×	×	0.0 ± 0.0

a All experiments used 3.2 mM glucose and a 3 mM concentration of the corresponding aromatic substrate as organic carbon sources.

b Strains are defined in Table 3.

c Yield reported as average ± standard deviation of results for three biological replicates.

d Results for strain 12444PDC are the same as reported in Table 2 and are included here to facilitate comparison.

Notably, in the two experiments in which we observed GA accumulation (Fig. 8A and C), the medium darkened in color, which could be due to a rapid nonenzymatic transformation of GA under the conditions used for these experiments. Since the sum of the molar concentration of unreacted syringic acid plus the accumulated metabolites does not add up to 100% in either experiment, these results also suggest that the GA produced was partially degraded either abiotically or biologically.

**FIG 9:**
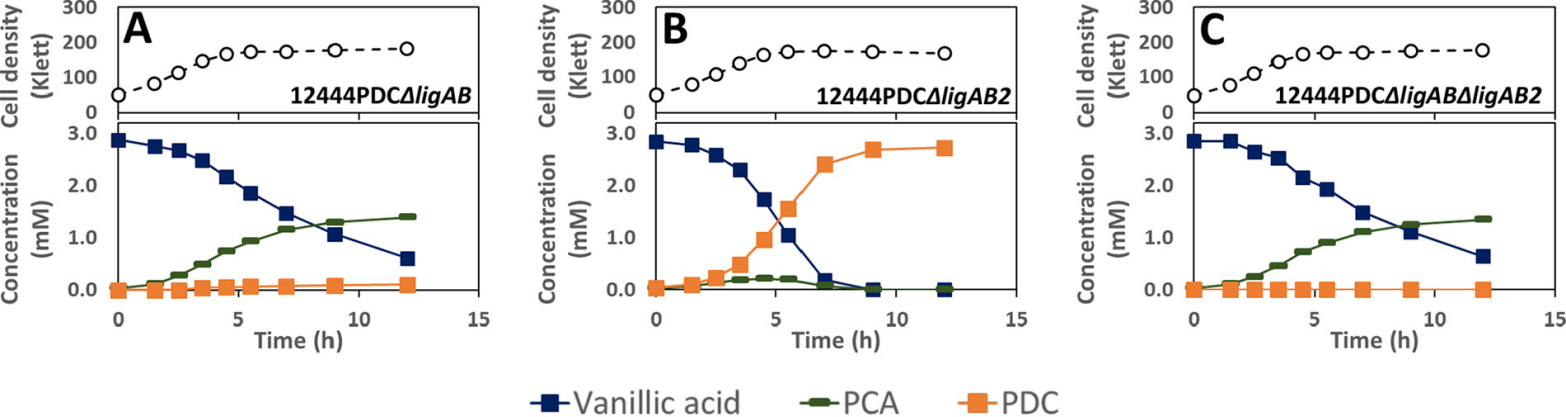
Growth and extracellular compound concentrations of *N. aromaticivorans* strains 12444PDC Δ*ligAB* (A), 12444PDC Δ*ligAB2* (B), and 12444PDC Δ*ligAB* Δ*ligAB2* (C) cultured in minimal medium supplemented with 3.2 mM glucose and 3 mM vanillic acid. All strains contain the Δ*1879* gene deletion. See Table 3 for the definition of strain names. Values correspond to the average results for three biological replicates. Error bars are included but in most cases not visible because they are smaller than the symbols in the plots. Abbreviations: PCA, protocatechuic acid; PDC, 2-pyrone-4,6-dicarboxylic acid.

The role of the putative ring-opening dioxygenases in vanillic acid degradation was also tested using the deletion mutants in the PDC-producing strain background (Fig. 9). Both the LigAB and LigAB2 mutant derivatives of the PDC-producing strain showed degradation of vanillic acid, but only the mutant with intact *ligAB* genes produced high yields of extracellular PDC (Fig. 9B; Table 4). The strains with deleted *ligAB* genes, 12444PDC Δ*ligAB* (Fig. 9A) and 12444PDC Δ*ligAB* Δ*ligAB2* (Fig. 9C), had a lower consumption of vanillic acid, removing only ∼80% of it from the medium and converting ∼48% of it into extracellular PCA over the course of the experiments. These observations suggest that LigAB has a significant role in PCA ring opening in *N. aromaticivorans*. Notably, strain 12444PDC Δ*ligAB* also converted ∼12% of the vanillic acid into PDC, whereas 12444PDC Δ*ligAB* Δ*ligAB2* did not accumulate any detectable PDC in the medium, suggesting that in the absence of LigAB, LigAB2 may also function in PCA ring opening, while the absence of both LigAB and LigAB2 completely eliminates the PCA ring-opening activity in *N. aromaticivorans*.

### (ii) Activity of LigAB and LigAB2 with aromatic substrates.

**FIG 10:**
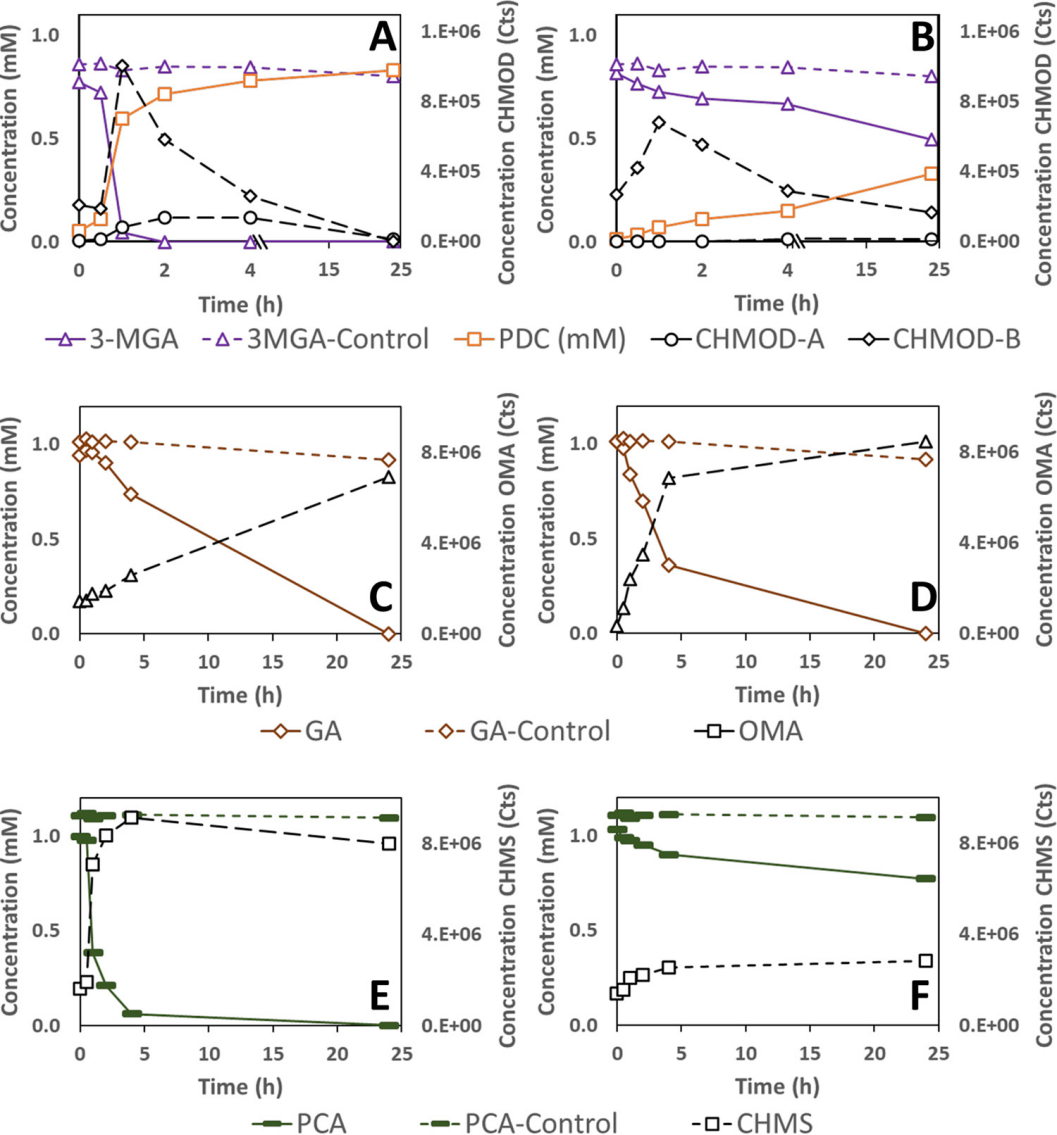
Substrate and product concentration of *in vitro* assays of LigAB (Saro_2812/3) (A, C, and E) and LigAB2 (Saro_1233/4) (B, D, and F) on 3-MGA (A and B), GA (C and D), and PCA (E and F). Concentrations of substrates in assays performed in the absence of added recombinant protein are shown by dashed lines and with the same symbol and color as that for the condition with enzyme. Samples were analyzed by HPLC-MS/MS, and concentrations correspond to the average results for three replicate assays. Abbreviations: 3-MGA, 3-methylgallate; PDC, 2-pyrone-4,6-dicarboxylic acid; CHMOD, 4-carboxy-2-hydroxy-6-methoxy-6-oxohexa-2,4-dienoate; GA, gallic acid; OMA, 4-oxalomesaconate; PCA, protocatechuic acid; CHMS, 4-carboxy-2-hydroxy-*cis,cis*-muconate-6-semialdehyde.

To investigate the predicted activities of LigAB and LigAB2 in aromatic ring opening, we purified recombinant forms of the proteins and tested them for activity *in vitro* (Fig. 10). With 3-MGA as the substrate, LigAB completely converted the 3-MGA from the reaction mixture within 2 h (Fig. 10A), whereas LigAB2 converted only ∼20% of the 3-MGA over the course of 24 h (Fig. 10B). With each purified enzyme, two products that transiently accumulated in the assay were identified as isomers of CHMOD (see supplemental material), and a third product was identified as PDC. These results show that LigAB catalyzes the transformation of 3-MGA to CHMOD and that CHMOD is subsequently transformed to PDC, as proposed previously (9). They also show that LigAB2 can also convert 3-MGA to CHMOD, albeit at a lower rate than with purified LigAB under the assay conditions used, which is consistent with results from the *in vivo* analyses of defined mutants in each of the corresponding genes (Fig. 7 and 8).

Both LigAB and LigAB2 also showed activity when tested with GA as the substrate (Fig. 10C and D). One compound that accumulated in these assays has been identified as OMA, the expected product of this ring-opening reaction (see supplemental material). Under identical assay conditions, LigAB2 degraded ∼31% of the GA after 2 h of reaction (Fig. 10D), whereas LigAB degraded only ∼11% (Fig. 10C). These findings suggest that of these two ring-opening dioxygenases, *N. aromaticivorans* LigAB2 has higher catalytic activity with GA than purified LigAB.

When tested with PCA as the substrate under identical assay conditions, LigAB converted ∼81% of the PCA from the reaction mixture after 2 h (Fig. 10E), while LigAB2 converted only ∼14% of the PCA over the same time span (Fig. 10F). The reaction product from both assays was identified as CHMS (see supplemental material), consistent with the expected ring-opening product of these enzymes with PCA as a substrate. The relative rates of PCA disappearance in assays using LigAB and LigAB2 suggest that under the assay conditions, LigAB is more catalytically active with PCA, which is consistent with the *in vivo* experiments with the individual mutant strains (Fig. 9).

## DISCUSSION

Strategies to successfully engineer efficient microbial catalysts that produce valuable compounds from chemically depolymerized lignocellulosic biomass have several requirements, including the need for the microorganisms to funnel a heterogenous mixture of plant-derived phenolic compounds through central pathways and the ability to genetically engineer the microorganisms to direct the flow of carbon from central aromatic metabolic pathways to the production of valuable compounds. To develop these strategies, it is necessary to acquire a detailed understanding of the native aromatic metabolic pathways in the microorganisms to be used as chassis for lignin valorization. This study focuses on advancing the knowledge of native aromatic metabolism in *N. aromaticivorans*, a sphingomonad of interest because it efficiently degrades the major S, G, and H phenolic substituents of plant biomass (9, 15) and because it is equipped with metabolic pathways for breaking down interunit linkages in lignin (17–20). The ability of *N. aromaticivorans* to efficiently degrade many aromatic compounds may be linked to having functionally redundant aromatic degradation pathways and enzymes with broad substrate specificity. While having redundant pathways and enzyme promiscuity may confer this microorganism with ecological advantages in nature, these features can create challenges or opportunities when engineering such microorganisms to produce high yields of a desired compound. For example, we reported earlier that the 12444PDC strain of *N. aromaticivorans* is able to funnel multiple lignin-derived aromatic compounds into PDC, but the PDC yields from S phenolics were lower than those from G and H phenolics (9). A possible explanation for this observation is pathway redundancy (16), which would allow *N. aromaticivorans* to channel a fraction of the S phenolics through one or more uncharacterized pathways that were not blocked in the 12444PDC strain.

**FIG 11:**
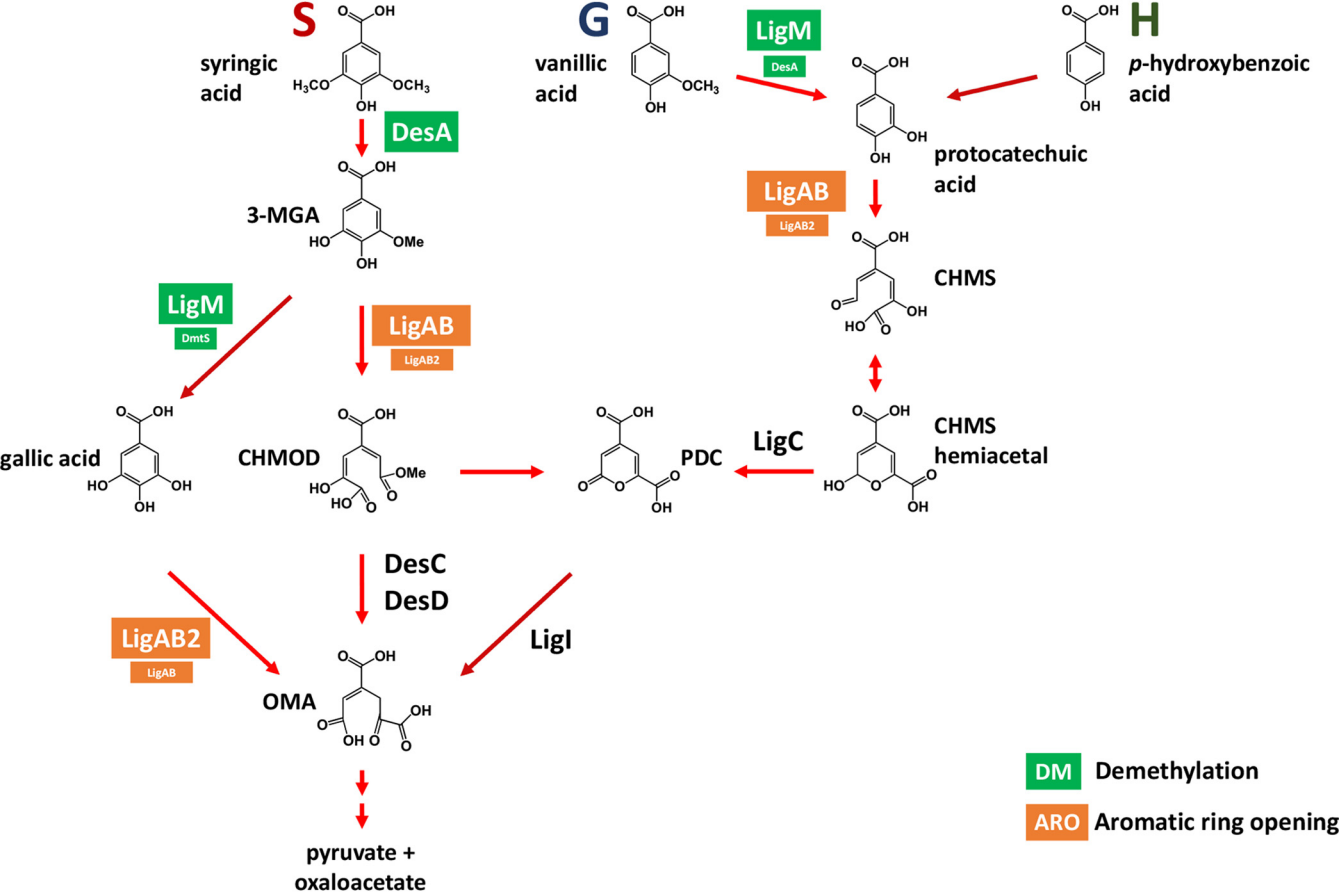
Updated pathway for metabolism of S, G, and H phenolics by *N. aromaticivorans*, with specific assignment of *O-*demethylase and aromatic ring-opening dioxygenase activities based on the *in vivo* and *in vitro* experiments described in this study. Enzymes previously identified whose functions are not yet verified *in vitro* are shown in black (9, 15) Abbreviations: 3-MGA, 3-methylgallate; CHMOD, 4-carboxy-2-hydroxy-6-methoxy-6-oxohexa-2,4-dienoate; CHMS, 4-carboxy-2-hydroxy-*cis,cis*-muconate-6-semialdehyde; PDC, 2-pyrone-4,6-dicarboxylic acid; OMA, 4-oxalomesaconate.

Based on the published analysis of aromatic metabolism of *Sphingobium* sp. SYK-6 (Fig. 1), we posited that one uncharacterized step might be the *O-*demethylation of 3-MGA to form GA with subsequent aromatic ring opening to produce OMA (Fig. 1). Because *O-*demethylation and aromatic ring opening are also involved in other branches of the aromatic metabolism pathways, in this work we systematically evaluated these two functions in *N. aromaticivorans*. Below we discuss the new information derived from the integration of *in vivo* experiments with mutant strains and *in vitro* experiments with purified enzymes (summarized in Fig. 11). The knowledge gained from these experiments helps us define roles for enzymes not previously described in *N. aromaticivorans* or other sphingomonads, identify functional pathway redundancy in the metabolism of S phenolics by this organism, and refine predictions of the substrate specificity of key *N. aromaticivorans* enzymes.

### *O-*Demethylation reactions.

The two H_4_folate-dependent *O-*demethylases, DesA and LigM, and the newly identified DmtS, a potential new *O*-demethylase, were each shown to have a role in the metabolism of S and G phenolics in *N. aromaticivorans* (Fig. 11). While we obtained genetic evidence for the role of DmtS as an *O-*demethylase, we were not able to purify recombinant DmtS, so we lack direct information about its substrate specificity. Of known *O-*demethylases, DmtS is most similar in its predicted amino acid sequence (∼27% identical) to the monooxygenase component (subunit A) of the vanillic acid *O-*demethylase VanAB, whose function has been demonstrated in other bacteria (29, 30) and that plays a key role in aromatic metabolism in P. putida (27, 28). In P. putida and other bacteria, the *vanA* gene is found in an operon with *vanB*, which encodes the putative reductase, VanB. However, *dmtS* is not contained in a gene cluster in *N. aromaticivorans* with a gene that encodes a protein with amino acid sequence identity to a known VanB reductase. Nevertheless, we were able to use mutants lacking *dmtS* to confirm its role in aromatic metabolism in *N. aromaticivorans*.

### (i) *O*-Demethylation of syringic acid.

Several of our results support the role of the H_4_folate-dependent *O-*demethylase DesA in the demethylation of syringic acid to 3-MGA. First, the *in vitro* assays with DesA showed stoichiometric conversion of syringic acid to 3-MGA, and the *in vitro* assays with LigM preparations that were active with other aromatic compounds showed no detectable activity with this substrate (Fig. 6). Second, *in vivo* experiments with the set of putative *O-*demethylase mutants showed that DesA was essential for *N. aromaticivorans* growth on syringic acid (Fig. 2). Third, the experiments with the set of putative *O-*demethylase mutants constructed in the previously reported PDC-producing strain (12444PDC) showed that all of the mutants lacking *desA* did not degrade syringic acid (Fig. 3).

To our knowledge, no enzymes other than DesA have been reported to demethylate syringic acid and produce 3-MGA in any sphingomonad. Indeed, in *Sphingobium* sp. SYK-6, DesA has been proposed to be the only enzyme responsible for syringic acid *O-*demethylation, and, similarly to *N. aromaticivorans*, the deletion of the *desA* gene prevents the mutant strain from growing on syringic acid as the sole substrate (25). In addition, deletion of the *desA* gene in strain 12444PDC completely blocks syringic acid degradation and PDC production in all the mutant strains even when cell growth is promoted by the addition of glucose in the growth medium.

### (ii) *O*-Demethylation of 3-MGA.

Although *O-*demethylation of 3-MGA to GA has been described in *Sphingobium* sp. SYK-6 (26), evidence for enzymes involved in this reaction in *N. aromaticivorans* was lacking before our studies. Here, we provide several lines of evidence that 3-MGA is converted to GA in *N. aromaticivorans*, but it is not the major route of 3-MGA metabolism. Instead, our results support the hypothesis that aromatic ring opening of 3-MGA to CHMOD is the major route for the metabolism of syringic acid. First, the nonstoichiometric conversion of syringic acid to PDC in the 12444PDC strain (Fig. 3A) is evidence for the existence of a secondary pathway that supports the degradation of a small fraction (∼15%) of the syringic acid in the PDC-producing strain. Second, stoichiometric conversion of syringic acid to PDC is achieved in the mutant that has an intact *desA* (necessary for the conversion of syringic acid to 3-MGA) and lacks both *ligM* and *dmtS* (Fig. 3G), leading to the hypothesis that both LigM and DmtS are active in this secondary pathway. This hypothesis was further confirmed in experiments using 3-MGA as the aromatic substrate (Fig. 4), where stoichiometric conversion of 3-MGA to PDC is achieved only by simultaneous deletion of *ligM* and *dmtS* (Table 2). Third, the *in vitro* assays with purified recombinant LigM (Fig. 6) showed that GA was stoichiometrically produced from 3-MGA. Finally, when comparing PDC yields from the 12444PDC strain and the strains with deletion of either *dmtS* or *ligM* (Table 2), a small but reproducible increase in PDC yield occurs when *ligM* is deleted, suggesting that *in vivo*, LigM is more active in 3-MGA demethylation than DmtS.

Based on a comparison of our results to that from other labs, the role of 3-MGA *O-*demethylation in the metabolism of syringic acid appears to be different in *N. aromaticivorans* and *Sphingobium* sp. SYK-6. For instance, inactivation of *ligM* in *N. aromaticivorans* did not cause a detectable effect in growth on syringic acid (Fig. 2A), whereas inactivation of this gene in *Sphingobium* sp. SYK-6 has a detrimental effect on both growth rate and final cell density when cells use syringic acid (25). This suggests that between the two functionally redundant pathways for converting 3-MGA to OMA in *N. aromaticivorans*, ring opening to CHMOD carries more flow of carbon than demethylation to GA, which is the opposite of what was reported for *Sphingobium* sp. SYK-6. In addition, our results also suggest redundancy in the enzymes that demethylate 3-MGA to GA in *N. aromaticivorans*, with both LigM and DmtS having this activity. *Sphingobium* sp. SYK-6 appears to have less enzyme redundancy in this reaction, as deletion of *ligM* eliminated 3-MGA conversion to GA in cell extract experiments (25).

### (iii) *O*-Demethylation of vanillic acid.

Based on our data, we propose that LigM plays a major role in demethylation of vanillic acid but that DesA can perform this reaction, although with reduced efficiency (Fig. 11). First, the *in vitro* assays showed that both of these enzymes could convert vanillic acid to PCA, with LigM having a higher degradation rate (Fig. 6). Second, *in vivo* experiments with the set of *O-*demethylase mutants in the wild-type background showed that all mutants with an intact *ligM* gene grew as well as the parent strain, strains lacking *ligM* but with intact *desA* showed a detectable growth defect, and strains lacking both *ligM* and *desA* could not grow in the presence of vanillic acid (Fig. 2). Consistent with these findings, our *in vivo* experiments with the set of mutants constructed in the 12444PDC background confirmed that deleting *ligM* lowered the rates of vanillic acid degradation, deleting *desA* alone did not have an effect, but that deleting both *ligM* and *desA* prevented vanillic acid degradation (Fig. 5). Finally, a role for DmtS in vanillic acid *O-*demethylation was not evident in the results of any of the experiments. These observations are consistent with the predicted role of LigM and DesA in the metabolism of vanillic acid by *Sphingobium* sp. SYK-6 (25).

### Aromatic ring-opening reactions.

Aromatic ring opening of phenolic compounds in sphingomonads is predicted to be catalyzed by extradiol dioxygenases (cleaving at the 4,5 position), which often exhibit broad substrate specificity (16, 31, 32). In *Sphingobium* sp. SYK-6, at least three dioxygenases are reported to be involved in the metabolism of S and G compounds: LigAB, with highest activity on PCA (33); DesZ, with highest activity on 3-MGA and GA (21); and DesB, specific for GA (24). In contrast, previous analysis of *N. aromaticivorans* has implicated only one extradiol dioxygenase, LigAB, in aromatic ring opening of phenolic compounds (15). In this study, we showed that two aromatic ring-opening enzymes in *N. aromaticivorans*, LigAB and LigAB2, catalyzed extradiol cleavage of 3-MGA, GA, and PCA, the three intermediates in the pathways for metabolism of S, G, and H aromatics whose rings are capable of being enzymatically opened by a 4,5-dioxygense (Fig. 11). In addition, our results show that LigAB has higher activity with 3-MGA and PCA than LigAB2, whereas LigAB2 has higher activity with GA than LigAB.

### (i) Aromatic ring opening of 3-MGA.

Our results support the hypothesis that LigAB is the primary enzyme for 3-MGA ring opening in *N. aromaticivorans* (15). They also provide new evidence that LigAB2 has activity with 3-MGA and might partially substitute for the role of LigAB.

The *in vitro* assays using recombinant LigAB and LigAB2 proteins with 3-MGA as a substrate showed accumulation and disappearance of the known stereoisomers of CHMOD and accumulation of PDC (Fig. 10A and B). *In vitro* accumulation of PDC from 3-MGA has also been observed in enzyme assays with the LigAB of *Sphingomonas* sp. SYK-6 (22). Rapid PDC accumulation in those experiments led to the proposal that PDC is a product of LigAB activity in *Sphingomonas* sp. SYK-6. Several results from our reactions of LigAB and LigAB2 from *N. aromaticivorans* with 3-MGA led us to the different conclusion that CHMOD is the direct product of the LigAB and LigAB2 reactions with 3-MGA and that CHMOD is nonenzymatically converted to PDC under the conditions of the assays. First, CHMOD is unstable in aqueous solution, and nonenzymatic cyclization of CHMOD to PDC at neutral pH has been reported (34). Unfortunately, the CHMOD appears to cyclize under our reaction conditions too fast for us to be able to purify this compound for determination of its molar concentration in our assays. However, the sum of the 3-MGA and PDC molar concentrations over the course of the enzymatic reactions inversely correlated with the accumulation of the CHMOD isomers (see Fig. S7 in the supplemental material), suggesting that CHMOD is an intermediate in the conversion of 3-MGA to PDC. Third, at the end of the LigAB reaction, when 3-MGA and CHMOD are not detectable (Fig. 10A), PDC accumulation reaches the initial concentration of 3-MGA in the assay, indicating the absence of any other potential products of CHMOD degradation. Finally, the results of *in vitro* assays of LigAB and LigAB2 with other substrates (Fig. 10) produced products consistent with the proposed function of these enzymes as extradiol aromatic ring-opening dioxygenases, and CHMOD is the expected product of such reaction when 3-MGA is the substrate (Fig. 11).

### (ii) GA ring opening.

The *in vitro* evidence obtained in this study indicates that LigAB2 reacts more rapidly with GA than with 3-MGA or PCA and confirms OMA as the product of GA ring opening (Fig. 10). The experiments with the set of deletion mutants in the 12444PDC background (Fig. 8) also provided evidence that either LigAB or LigAB2 might be responsible for metabolizing the fraction of syringic acid that is normally channeled through GA in the 12444PDC strain. However, under the conditions of our studies, the GA that accumulated in the medium apparently converted abiotically to an unknown product. Thus, further research is needed to specifically ascertain the role of LigAB and LigAB2 on GA transformation *in vivo*.

### (iii) PCA ring opening.

The results of *in vitro* (Fig. 10) and *in vivo* (Fig. 9) experiments with vanillic acid provided evidence that the ring opening of PCA is catalyzed primarily by LigAB but that LigAB2 could partially substitute *in vivo* when *ligAB* was deleted. Furthermore, deleting both sets of genes eliminated growth (Fig. 7) and PDC production (Fig. 9), indicating that no other enzyme in *N. aromaticivorans* could catalyze the ring opening of PCA under our growth and medium conditions.

### Implications for PDC production from lignin-derived aromatics by *N. aromaticivorans*.

We have previously tested PDC production from plant-derived phenolics by *N. aromaticivorans* (9), because this native pathway intermediate is a potential building block for bio-based plastic and epoxy adhesives (35). We showed that strain 12444PDC, which contains mutations that block the conversion of PDC and CHMOD to OMA, could transform S, G, and H phenolics to PDC but exhibited lower PDC yields from S phenolics than from G or H phenolics (9). Based on this observation, we proposed that strain 12444PDC contained additional, previously unidentified, gene products that could metabolize S phenolics (9). In this study, we identified these previously unknown enzymes in the proposed alternative pathway for metabolism of syringic acid in *N. aromaticivorans* that *O-*demethylates 3-MGA to GA (Fig. 11). We further showed that these same enzymes catalyze ring opening of GA to OMA (Fig. 11). The activity of the enzymes in this previously unknown alternative pathway helps explain why strain 12444PDC had lower PDC yields from S phenolics, since our data suggest that ∼15% of the 3-MGA follows the GA-OMA route and does not contribute to PDC production in strain 12444PDC. Although deleting the genes in this alternative pathway might increase the yield of PDC, the promiscuity of the other *N. aromaticivorans O-*demethylases described in this study implies that single gene deletions may not be sufficient to maximize PDC yields. As predicted, stoichiometric production of PDC from either syringic acid (Fig. 3G) or 3-MGA (Fig. 4G) was possible only with the deletion of both *ligM* and *dmtS* from the 12444PDC background. However, since *O-*demethylases are also needed in the demethylation of G phenolics, a potential side effect of *ligM* deletion (the main enzyme we found to be responsible for vanillic acid demethylation) could be the reduced conversion of G phenolics into PDC. Indeed, we observed the predicted negative effect in the rate of vanillic acid conversion when *ligM* was deleted (Fig. 5C), although the PDC yield was not affected when this gene was inactivated in the 12444PDC strain (Table 2). Since DesA was shown to have activity with vanillic acid, one potential strategy to compensate for the absence of LigM might be to engineer PDC-producing strains that overexpress *desA*, as has been done with overexpression of *vanAB* in P. putida to accelerate vanillic acid degradation (36).

The results of our studies also make a new prediction that deleting enzymes responsible for the aromatic ring-opening step does not appear to be a productive strategy for reducing the flow of substrates through this alternative *N. aromaticivorans* pathway due to the promiscuity of these dioxygenases. For example, while *in vitro* assays with the newly discovered LigAB2 showed its preference for GA over 3-MGA and PCA, its inactivation did not produce measurable effects on PDC yield (Table 4). Furthermore, inactivation of both LigAB and LigAB2 would also prevent aromatic ring opening with other biomass-derived phenolic substrates. From our data, we propose that the main function of LigAB2 *in vivo* is in ring-opening reactions of aromatic compounds other than S, G, and H phenolics. While *Sphingobium* sp. SYK-6 has a predicted homologue of *N. aromaticivorans* LigAB2 (encoded by SLG_37520 and SLG_37530), we are not aware of any studies that investigate its role in S, G, or H aromatic metabolism.

In conclusion, we identified a previously unknown alternative route for syringic acid catabolism, described new enzymes that are involved in this pathway (DmtS and LigAB2), and performed a systematic genetic and enzymatic analysis of the *O-*demethylases and aromatic ring-opening dioxygenases that function in *N. aromaticivorans*. The studies revealed that these *O-*demethylases and dioxygenases have activity with multiple substrates, suggesting that they are able to participate in different reactions within individual pathways (Fig. 11). The new knowledge on the metabolism of aromatic compounds by *N. aromaticivorans* obtained in this work has allowed us to design a strain (12444PDC Δ*ligM* Δ*dmtS*) with increased yield of PDC from syringic acid. It also will enable better predictions of metabolic routes that will facilitate engineering strains for improved yields of other desirable products from biomass-derived aromatics. While the interruption of 3-MGA *O-*demethylation (by deleting *ligM* and *dmtS*) in strain 12444PDC resulted in stoichiometric PDC production from syringic acid and vanillic acid, this new strain exhibited a reduced rate of vanillic acid degradation. This observation illustrates additional steps in aromatic acid degradation that are targets for future strain improvement.

## MATERIALS AND METHODS

### Bacterial strains, growth media, and culturing conditions.

Two variants of *N. aromaticivorans* DSM 12444, strain 12444 Δ*1879*, which lacks the gene Saro_1879 (putative *sacB*; Saro_RS09410) (17), and strain 12444 Δ*ligI* Δ*desCD* (also called 12444PDC), which lacks the genes Saro_1879, Saro_2819 (Saro_RS14300), Saro_2864 (Saro_RS14525), and Saro_2865 (Saro_RS14530) (9), were used as parent strains to generate the mutant strains shown in Table 1.

All genetic modifications were carried out using a variant of the plasmid pK18*mobsacB* (37), which contains *sacB* and a kanamycin resistance gene. The gene deletions were performed as previously described (9), with the details of the processes used here described in the supplemental material. All primers, plasmids, and Escherichia coli strains used for cloning and protein expression are listed in Tables 5, 6, and 7, respectively.

**TABLE 5:** Primers used in this study

Name	Sequence	Note(s)
Saro_2861-pK18_Amp-R	5′-**GTTTCTGCGGACTGGCTTTCTAGATGTTC**CG TTGACGTGCATGTCTGTCCTCTC-3′	Region in bold matches sequence in pK18msB-MCS1
Saro_2861_Del-F	5′-**CTTCTCGTTGTACGAGTAGCCG**GACTGACT CTCCCGACTTGAAAATGG-3′	Region in bold matches sequence in Saro_2861_Del-R
Saro_2861_Del-R	5′-**CAAGTCGGGAGAGTCAGTC**CGGCTACTCGT ACAACGAGAAGCAG-3′	Region in bold matches sequence in Saro_2861_Del-F
Saro_2861-pK18_Amp-F	5′-**CGATTCATTAATGCAGCTGGCACGACAG**G ACGAGATCAGCCTTTAGCCGATCC-3′	Region in bold matches sequence in pK18msB-MCS1
Saro_2404-pK18_Amp-R	5′-**GTTTCTGCGGACTGGCTTTCTAGATGTT**CCC CCACCCGTTTACTTCTTCATGC-3′	Region in bold matches sequence in pK18msB-MCS1
Saro_2404_Del-F	5′-**GCATCGGGAGAGCTTGAG**CAAGAAGTAAG GCTACAGGTGCGAACC-3′	Region in bold matches sequence in Saro_2404_Del-R
Saro_2404_Del-R	5′-**CACCTGTAGCCTTACTTCTTG**CTCAAGCTCT CCCGATGCGATTTC-3′	Region in bold matches sequence in Saro_2404_Del-F
Saro_2404-pK18_Amp-F	5′-**CGATTCATTAATGCAGCTGGCACGACAG**G CAATCTCGGAACTCGGCATCTACC-3′	Region in bold matches sequence in pK18msB-MCS1
Saro_1872-pK18_Amp-R	5′-**GTTTCTGCGGACTGGCTTTCTAGATGTT**CCA TGGGGTTTCCTTACTTGTACTTGC-3′	Region in bold matches sequence in pK18msB-MCS1
Saro_1872_Del-F	5′-**CTATCTCTGAGGGCAGTGGC**GAATTCGCGT TCGAGCAGGAAGACATG-3′	Region in bold matches sequence in Saro_1872_Del-R
Saro_1872_Del-R	5′-**CTTCCTGCTCGAACGCGAATTC**GCCACTGCC CTCAGAGATAGAGAG-3′	Region in bold matches sequence in Saro_1872_Del-F
Saro_1872-pK18_Amp-F	5′-**CGATTCATTAATGCAGCTGGCACGACAG**GA CAAGGCAGAAATCGTCGATGTGC-3′	Region in bold matches sequence in pK18msB-MCS1
Saro_2812/3-pK18_Amp-R	5′-**GTTTCTGCGGACTGGCTTTCTAGATGTTC**G CAAGACCATAGAGTTCAAACTTGAGAG-3′	Region in bold matches sequence in pK18msB-MCS1
Saro_2812/3_Del-F	5′-**CTACTCCTGTCTGGTCAG**CTACAGGCCCCTC TCCTTCA GC-3′	Region in bold matches sequence in Saro_2812/3_Del-R; underlined bases were modified from the genomic sequence to inactivate the Saro_2813 start codon and maintain the Saro_2814 stop codon
Saro_2812/3_Del-R	5′-**GAAGGAGAGGGGCCTGTAG**CTGACCAGAC AGGAGTAGTACCCATG-3′	Region in bold matches sequence in Saro_2812/3_Del-F
Saro_2812/3-pK18_Amp-F	5′-**CGATTCATTAATGCAGCTGGCACGACAG**GT GCATTCAATTCATTCGTCTTTGCGATGAG-3′	Region in bold matches sequence in pK18msB-MCS1
Saro_1233/4-pK18_Amp-R	5′-**GTTTCTGCGGACTGGCTTTCTAGATGTTC**G TGGTCCTCCTCGACATCAACATGC-3′	Region in bold matches sequence in pK18msB-MCS1
Saro_1233/4_Del-F	5′-**GTGTTGCATATGAAATGTCCGTC**CACGAGG TCAGCCGGAACTACCATATC-3′	Region in bold matches sequence in Saro_1233/4_Del-R
Saro_1233/4_Del-R	5′-**GTTCCGGCTGACCTCGTG**GACGGACATTTC ATATGCAACACATACGAATTTTCC-3′	Region in bold matches sequence in Saro_1233/4_Del-F
Saro_1233/4-pK18_Amp-F	5′-**CGATTCATTAATGCAGCTGGCACGACAG**CA GCTCTGTGTTGTAGCGTTGCTGTC-3′	Region in bold matches sequence in pK18msB-MCS1
Saro_2861_pVP-F	5′-**CTAACTTTGTTATTTTCGGCTTTCTG**GGATC CTCTGTTGATGAAACCGGTC-3′	Region in bold matches sequence in pVP302K
Saro_2861_pVP-R	5′-**GTATTTTCAGAGCGCGATCGCAGGA**ATGG CGGCAAAGAACCTCGAAGAG-3′	Region in bold matches sequence in pVP302K
Saro_2404_pVP-F	5′-**GTATTTTCAGAGCGCGATCGCAGGA**ATGTG CCAGACCCTAGAGCAGGTC-3′	Region in bold matches sequence in pVP302K
Saro_2404_pVP-R	5′-**CTAACTTTGTTATTTTCGGCTTTCTG**GGTTC GCACCTGTAGCCTTACTTCTTG-3′	Region in bold matches sequence in pVP302K
Saro_2813-2_pVP-HiFi_start	5′-**GTATTTTCAGAGCGCGATCGCAGGA**GTGA CTGACAACAGCTCGACCGATAAG	Region in bold matches sequence in pVP302K
Saro_2813-2_pVP-HiFi_stop	5′-**CTAACTTTGTTATTTTCGGCTTTCTG**GTACT ACTCCTGTCTGGTCAGTCAGTCC	Region in bold matches sequence in pVP302K
Saro_1233/4_pVP-F	5′-**GTATTTTCAGAGCGCGATCGCAGGA**ATGAC ACCTGAAGGAAACCGCGAG-3′	Region in bold matches sequence in pVP302K
Saro_1233/4_pVP-R	5′-**CTAACTTTGTTATTTTCGGCTTTCTG**CAGCA TATGTGGCGGAGCCGTC-3′	Region in bold matches sequence in pVP302K

**TABLE 6:** Plasmids used in this study

Plasmid	Details	Reference or source
pK18*mobsacB*	pMB1ori *sacB kanR mobT oriT*(RP4) *lacZ*α	37
pVP302K	*lac* promoter *lacI rtxA* (*Vibrio cholerae*) *kanR*; coding sequences for 8×His tags, TEV protease cleavage site	19
pRARE2	p15a ori *camR*; tRNA genes for 7 rare codons in E. coli	Novagen
pK18msB-MCS1	pK18*mobsacB* lacking the multiple cloning site, with a new XbaI site introduced	17
pK18msB/ΔSaro2861	pK18msB-MCS1 containing genomic regions flanking Saro_2861	This study
pK18msB/ΔSaro2404	pK18msB-MCS1 containing genomic regions flanking Saro_2404	This study
pK18msB/ΔSaro1872	pK18msB-MCS1 containing genomic regions flanking Saro_1872	This study
pK18msB/ΔSaro2812/3	pK18msB-MCS1 containing genomic regions flanking Saro_2812/3	This study
pK18msB/ΔSaro1233/4	pK18msB-MCS1 containing genomic regions flanking Saro_1233/4	This study
pVP302K/Saro2861	pVP302K containing *ligM* downstream of coding sequences for 8×His tag and TEV protease cleavage site	This study
pVP302K/Saro2404	pVP302K containing *desA* downstream of coding sequences for 8×His tag and TEV protease cleavage site	This study
pVP302K/Saro2812/3	pVP302K containing *ligAB* downstream of coding sequences for 8×His tag and TEV protease cleavage site	This study
pVP302K/Saro1233/4	pVP302K containing *ligA2B2* downstream of coding sequences for 8×His tag and TEV protease cleavage site	This study

**TABLE 7:** E. coli strains used in this study

Strain	Relevant characteristics	Reference or source
DH5α	F^−^ ϕ80 *lacZ*ΔM15 Δ(*lacZYA-argF*)*U169 recA1 endA1 hsdR17*(r_K_^−^ m_K_^+^) *phoA supE44* λ^−^ *thi-1 gyrA96 relA1*	Bethesda Research Laboratories
S17-1	*recA pro hsdR* RP4-2-Tc::Mu-Km::Tn*7*	42
B834	F^−^ *hsdS metE gal ompT*	38, 39

E. coli cultures were grown in LB medium containing 50 μg ml^−1^ kanamycin at 37°C. *N. aromaticivorans* cultures were grown in SMB medium (see reference 17 for recipe) supplemented with the indicated carbon source at 30°C. For routine culture and storage, SMB medium was supplemented with 1 g liter^−1^ glucose. For constructing mutants, SMB medium was supplemented with 1 g liter^−1^ glucose and either 50 μg ml^−1^ kanamycin or 100 g liter^−1^ sucrose as necessary. Cell density was monitored using a Klett-Summerson photoelectric colorimeter with a red filter.

### *N. aromaticivorans* growth experiments.

*N. aromaticivorans* cultures were grown overnight in SMB medium supplemented with 1 g liter^−1^ glucose. Cultures were diluted 1:1 with fresh medium containing 1 g liter^−1^ glucose and incubated for 1 h to resume cell growth. Cells from 2 ml of the growing culture were pelleted (5 min at 2,300 × *g*), washed with 1 ml SMB medium without carbon, and then resuspended into 600 μl SMB medium with no added carbon. Growth experiments were initiated by adding 80 μl of the cell suspension into 8 ml of fresh SMB medium supplemented with either 3 mM syringic acid or 3 mM vanillic acid. Cultures were grown aerobically in 20-ml test tubes, with shaking at 200 rpm at 30°C. Each experiment was repeated 3 times.

### *N. aromaticivorans* extracellular metabolite analysis.

Bacterial cell cultures were grown overnight in 20 ml of SMB medium supplemented with 1 g liter^−1^ glucose and then reactivated by adding 2 ml of fresh medium containing 1 g liter^−1^ glucose and incubated for 1 h. Experiments were initiated by inoculating 5 ml of active culture into 15 ml of fresh SMB medium supplemented with 5 mM glucose and either 4 mM syringic acid, 4 mM 3-MGA, or 4 mM vanillic acid. Cultures were grown aerobically in 125-ml conical growth flasks, with shaking at 200 rpm at 30°C. Samples were collected periodically, filtered (through 0.22-μm pores) to remove cells, and immediately analyzed by high-performance liquid chromatography–mass spectrometry (HPLC-MS) to monitor the extracellular aromatic compounds. Each experiment was repeated 3 times. PDC yields were calculated as the amount of PDC produced divided by the amount of aromatic substrate consumed. Comparison of PDC yields was done with an unpaired, two-tailed *t* test, with a *P* of ≤0.05 taken as a statistically significant difference in the observed yields.

### Recombinant enzyme expression and purification.

Genes Saro_2812/3 (*ligAB*), Saro_1233/4 (*ligAB2*), Saro_2861 (*ligM*), and Saro_2402 (*desA*) from *N. aromaticivorans* were independently cloned into plasmid pVP302K (19), which incorporates a His_8_ tag to the N terminus of the translated transcripts connected via a tobacco etch virus (TEV) protease recognition site (see the supplemental material for plasmid construction details). The expression plasmids were transformed into E. coli B834 (38, 39) containing plasmid pRARE2 (Novagen, Madison, WI), and transformants were grown in ZYM-5052 autoinduction medium (40) containing 50 μg ml^−1^ kanamycin and 20 μg ml^−1^ chloramphenicol. Recombinant proteins were purified by passing crude E. coli lysates through a nickel-nitrilotriacetic acid (Ni^2+^-NTA) column as described previously (17). His_8_ tags were cleaved from recombinant proteins using TEV protease, and proteins were passed again though a Ni-NTA column to remove the cleaved His_8_ tag and the TEV protease (which contains its own His tag).

### *In vitro* aromatic ring-opening dioxygenase assays.

Preliminary experiments with recombinant LigAB (Saro_2812/3) and LigAB2 (Saro_1233/4) purified in the presence of air suggested that the enzymes were catalytically inactive, as reported for other homologues of these proteins (41). We thus separately mixed LigAB and LigAB2 with reactivation buffer (buffer A, containing 20 mM Na_2_HPO_4_, 20 mM KH_2_PO_4_, 1 mM Fe_2_SO_4_, and 1 mM ascorbic acid and prepared anaerobically) to a concentration of 2 μM enzyme active sites under anaerobic conditions and incubated them anaerobically at 30°C for 21 h to reactivate the enzymes. Three solutions containing buffer B (20 mM Na_2_HPO_4_, 20 mM KH_2_PO_4_, and 1 mM ascorbic acid) and either 2 mM 3-MGA, 2 mM PCA, or 2 mM GA were prepared in the presence of air. Enzyme assays were performed in 2-ml polypropylene vials inside an anaerobic chamber at 30°C by mixing 750 μl of reactivated enzyme in buffer A with 750 μl of aromatic substrate in buffer B. As a control, we mixed 750 μl of buffer A without enzyme with 750 μl of each aromatic substrate in buffer B. After 30 min, the vials were exposed to the ambient atmosphere outside the anaerobic chamber for 10 min to expose the reactions to the O_2_ predicted to be a substrate for the ring-opening reaction and then transferred back into the anaerobic chamber. Exposure to O_2_ was repeated at 2 h and 4 h after the assay was initiated. Samples (250 μl) were collected at time zero, at 30 min, and at 1, 2, 4, and 24 h, immediately mixed with 50 μl of 1 N HCl to terminate the reaction, and then analyzed by HPLC-MS to quantify substrate disappearance and formation of any products. Identification of OMA, CHMOD, and PDC was performed by gas chromatography (GC)-MS of samples extracted with ethyl acetate immediately after collection. CHMS was converted into 2,4-pyridinedicarboxylic acid (PDCA) by the addition of (NH_4_)_2_SO_4_ (33), which was then identified by HPLC/UV. Assays were performed in triplicate.

### Aromatic *O-*demethylase enzyme assays.

Enzyme assays were performed in triplicate under anaerobic conditions at 30°C (because of the expected O_2_ sensitivity of H_4_folate). Recombinant LigM (Saro_2861) and DesA (Saro_2404) were separately mixed with buffer C (20 mM Na_2_HPO_4_, 20 mM KH_2_PO_4_, and 1 mM or 2 mM H_4_folate; prepared anaerobically) to concentrations of 2 μM enzyme active sites under anaerobic conditions. Three independent solutions containing buffer D (20 mM Na_2_HPO_4_ and 20 mM KH_2_PO_4_) and either 1 mM 3-MGA, 1 mM PCA, or 1 mM GA were prepared aerobically. Enzyme reactions were initiated in 2-ml polypropylene vials inside an anaerobic chamber at 30°C by mixing 750 μl of an enzyme in buffer C with 750 μl an aromatic substrate in buffer D. A control was run by mixing 750 μl of buffer C without enzyme with 750 μl each aromatic substrate in buffer D. Samples (250 μl) were collected at time zero, at 30 min, and at 1, 2, 4, and 24 h, immediately mixed with 50 μl of 1 N HCl to stop the reaction, and then analyzed by HPLC-MS to quantify substrate disappearance and product formation.

### Analysis of extracellular metabolites and enzyme reaction products.

All culture supernatants and *in vitro* enzyme assay samples were filtered (0.2 μm) prior to chemical analysis. Quantitative analyses of aromatic compounds were performed on a Shimadzu triple-quadrupole liquid chromatography mass spectrometer (LC-MS) (Nexera XR HPLC-8045 MS/MS). Reverse-phase HPLC was performed using a binary gradient mobile phase consisting of solvent A (0.2% formic acid in water) and methanol (gradient profile shown in Fig. S8 in the supplemental material) and a Kinetex F5 column (2.6-μm pore size, 2.1-mm inside diameter, 150-mm length; product no. 00F-4723-AN). All compounds were detected by multiple-reaction monitoring (MRM) and quantified using the strongest MRM transition (Table S2).

Identification of OMA and PDC was performed by GC-MS. Sample aliquots (150 μl) were acidified with HCl to pH <2 and extracted with ethyl acetate (3 times, 500 μl each time). The three extraction samples were combined, dried under a stream of N_2_ at 40°C, derivatized by the addition of 150 μl of pyridine and 150 μl of *N,O*-bis(trimethylsilyl)trifluoroacetamide with trimethylchlorosilane (99:1 [wt/wt]; Sigma), and incubated at 70°C for 45 min. The derivatized samples were analyzed on an Agilent GC-MS (GC model 7890A; MS model 5975C) equipped with a (5% phenyl)-methylpolysiloxane capillary column (Agilent model HP-5MS). The injection port temperature was held at 280°C, and the oven temperature program was held at 80°C for 1 min and then ramped at 10°C min^−1^ to 220°C, held for 2 min, ramped at 20°C min^−1^ to 310°C, and held for 6 min. The MS used an electron impact (EI) ion source (70 eV) and a single-quadrupole mass selection scanning at 2.5 Hz, from 50 to 650 *m/z*. The data were analyzed with the Agilent MassHunter software suite.

The product of PCA aromatic ring opening, predicted to be CHMS, was analyzed by its conversion into 2,4-pyridinedicarboxylic acid (PDCA). One hundred microliters of sample was pH neutralized by the addition of 10 μl of a 1.67 N solution of NaOH. In addition, 5 μl of a 10% solution of (NH_4_)_2_SO_4_ was added and then incubated at room temperature for 24 h. Samples were analyzed by HPLC-UV using the same HPLC conditions described above. Eluent was analyzed for light absorbance between 190 and 400 nm with a Shimadzu SPD-M20A spectrophotometer.

### Chemicals.

All SMB medium reagents, gallic acid, and vanillic acid were purchased from Sigma-Aldrich (St. Louis, MO). Syringic acid was purchased from TCI (Tokyo Chemical Industry)-America (Portland, OR). 3-MGA was purchased from Carbosynth (Berkshire, UK). Protocatechuic acid was purchased from Sigma-Aldrich (St. Louis, MO).

## Supplementary Material

Supplemental file 1
