# Supplementary material for: Redundancy in Aromatic O-Demethylation and Ring-Opening Reactions in Novosphingobium aromaticivorans and Their Impact in the Metabolism of Plant-Derived Phenolics
Source: Appl Environ Microbiol. 2021 Mar 26;87(8):e02794-20. doi: 10.1128/AEM.02794-20 (PMC8091115; doi:10.1128/AEM.02794-20)
Supplement: Supplemental file 1 [file AEM.02794-20-s0001.pdf]

Electronic Supplementary Information

Redundancy in aromatic O-demethylation and ring opening reactions in *Novosphingobium aromaticivorans* and their impact in the metabolism of plant derived phenolics

Jose M. Perez,[a,b,c] Wayne S. Kontur,[b,c] Carson Gehl,[b,c,d] Derek M. Gille,[b,c] Yanjun Ma,[b,c] Alyssa V. Niles,[b,c] German Umana,[b,c] Timothy J. Donohue,[b,c,e] and Daniel R. Noguera[a,b,c]*

[a]Department of Civil and Environmental Engineering, University of Wisconsin-Madison, Madison, WI, USA.

[b]DOE Great Lakes Bioenergy Research Center, Madison, WI, USA

[c] Wisconsin Energy Institute, University of Wisconsin-Madison, Madison, WI, USA

[d]Department of Biomedical Engineering, University of Wisconsin-Madison, Madison, WI, USA

[e]Department of Bacteriology, University of Wisconsin-Madison, Madison, WI, USA

*Corresponding author: Daniel R. Noguera, dnoguera@wisc.edu

Supplementary Information

Construction of plasmids for generating in-frame deletions of Saro_2861, Saro_2404, Saro_1872, Saro_2812/3, or Saro_1233/4. Regions of *N. aromaticivorans* genomic DNA containing ~1100 bp upstream and downstream of Saro_2861, Saro_2404, Saro_1872, Saro_2812/3, or Saro_1233/4 were PCR amplified separately using the pairs of primers Saro_2861-pK18_Amp-R/Saro_2861_Del-F, Saro_2861-pK18_Amp-F/Saro_2861_Del-R, Saro_2404-pK18_Amp-R/Saro_2404_Del-F, Saro_2404-pK18_Amp-F/Saro_2404_Del-R, Saro_1872-pK18_Amp-R/Saro_1872_Del-F, Saro_1872-pK18_Amp-F/Saro_1872_Del-R, Saro_2812/3-pK18_Amp-R/Saro_2812/3_Del-F, Saro_2812/3-pK18_Amp-F/Saro_2812/3_Del-R, Saro_1233/4-pK18_Amp-R/Saro_1233/4_Del-F, and Saro_1233/4-pK18_Amp-F/Saro_1233/4_Del-R (Table S1). The pairs of DNA amplified flanking regions for each gene were combined with linearized pK18*msB*-MCS1 (1) using NEBuilder® HiFi DNA Assembly Master Mix (New England Biolabs, Ipswich, MA) to produce the plasmids pK18msB/ΔSaro2861, pK18msB/ΔSaro2404, pK18msB/ΔSaro1872, pK18msB/ΔSaro2812/3, and pK18msB/ΔSaro1233/4, respectively. The plasmids were then transformed into NEB 5-alpha competent *E. coli* (New England Biolabs). The transformed *E. coli* cells were then cultured in LB media + kanamycin, the plasmids purified using a Quiagen® Plasmid Maxi Kit (Qiagen, Germany), and DNA sequencing was used to confirm the presence of the desired deletion.

Deletion of Saro_2861, Saro_2404, Saro_1872, Saro_2812/3, or Saro_1233/4 from *N. aromaticivorans*. The plasmids constructed above were individually transformed into competent *E. coli* S17-1 and subsequently mobilized into different strains of *N. aromaticivorans* (see Table 1) via bacterial conjugation. Transconjugant cells of *N. aromaticivorans* (single cross overs) were isolated on SMB plates containing 1g/L glucose and 50ug/mL kanamycin. To generate and isolate cells that subsequently eliminated the plasmid via a second instance of homologous recombination (double crossovers), single

crossover cells were cultured on solid SMB media containing 1g/L glucose and 10% sucrose. PCR amplified regions of the target genes were sequenced to verify the colonies in which the desired genes were deleted.

Construction of protein expression plasmids. Saro_2861, Saro_2404, Saro_2812/3, and Saro_1233/4 were individually amplified from *N. aromaticivorans* DSM12444 genomic DNA with the pairs of primers Saro_2861_pVP-F and Saro_2861_pVP-R, Saro_2404_pVP-F and Saro_2404_pVP-R, Saro_2813-2_pVP-HiFi_start and Saro_2813-2_pVP-HiFi_stop, and Saro_1233/4_pVP-F and Saro_1233/4_pVP-R, respectively (Table S3). Each resulting DNA fragment was combined with linearized pVP302K (2) using NEBuilder® HiFi DNA Assembly Master Mix, as previously described (3), generating plasmids pVP302K/Saro2861, pVP302K/Saro2404, pVP302K/Saro2812/3, and pVP302K/Saro1233/4, which consist of a T5 promoter followed by coding sequences for a His_8-tag, a tobacco etch virus (TEV) protease recognition site, and the coding sequence of the *N. aromaticivorans* gene(s).

Identification of products from experiments with recombinant LigAB and LigAB2

To investigate the activities of LigAB and LigAB2 and identify reaction products using different substrates (3-methoxygallic acid (3-MGA), gallic acid (GA), and protocatechuic acid (PCA)), we purified recombinant forms of the proteins and tested them for activity *in vitro*. We know from studies with homologs of these enzymes that they are prone to inactivation by oxidation of an active site Fe^{2+} (4). To minimize O_2 inactivation, while also providing the O_2 that is expected to be one of the enzyme substrates, we performed these assays using a hybrid anaerobic/aerobic method consisting of exposing the reaction vials to air for periods of 5 min after 10 min, 30 min, 2 h, and 4 h have elapsed in the reaction. Samples (250 µL) were collected before adding enzyme, at indicated times, and at 24 h after reaction initiation, and mixed with 50 µL 1N HCl to inactivate the enzyme before analysis. For assays using 3-MGA or GA as a substrate, samples were analyzed by HPLC-MS to monitor disappearance of the substrate and the formation of CHMOD, PDC, and OMA. GC-MS was used to verify the identity of PDC and OMA. For assays using PCA, all samples were analyzed by HPLC-MS to monitor the substrate disappearance and the formation of CHMS. To verify the identity of CHMS, it was converted into 2,4-pyridinedicarboxylic acid (PDCA) and analyzed by HPLC-UV. Its light absorbance spectrum was compared with authentic PDCA.

When tested with 3-MGA as the substrate, HPLC-MS analysis showed the transient accumulation of 2 compounds with m/z = 215 in the negative mode, which is consistent with them being stereoisomers of CHMOD (MW = 216.15). GC-MS analysis of the TMS-derivatized ethyl acetate extracts of samples collected after 1 hour of reaction, only showed the presence of TMS-PDC (Figures S2 and S3), suggesting that any remaining CHMOD present in the samples cyclized into PDC during sample storage and the derivatization process before analysis.

When tested with GA as the substrate, HPLC-MS analysis showed the accumulation of a compound with a m/z = 201 in the negative mode, which is consistent with it being OMA (MW = 202.12). GC-MS analysis of the TMS-derivatized ethyl acetate extracts of samples collected after 24 hour of reaction showed a compound with a fragmentation pattern identical to that previously found for the TMS derivative of the enol form of OMA (5) (Figures S4 and S5). This analysis also showed the presence of a TMS derivative of PDC, attributed to the production of PDC from the reaction of OMA with HCl, which was added to quench the reaction (6).

When tested with PCA as the substrate, HPLC-MS analysis showed the accumulation of a compound with a m/z = 185 in the negative mode, which is consistent with it being CHMS (MW = 186.12). Samples collected after 24 hours of reaction and treated with $(NH_4)_2SO_4$ were analyzed by HPLC-UV. Samples from *in vitro* assays with LigAB showed the presence of a peak with identical retention time and light absorbance spectrum (Figures S6C and S6D) of that of authentic PDCA (Figures S6A and S6B). Samples from *in vitro* assays with LigAB2 showed a peak similar to PDCA and a peak corresponding to unreacted PCA (Figures S6E and S6F). Samples from control condition without enzyme only showed the presence of PCA (Figure 6G).

Supplemental Figures and Tables

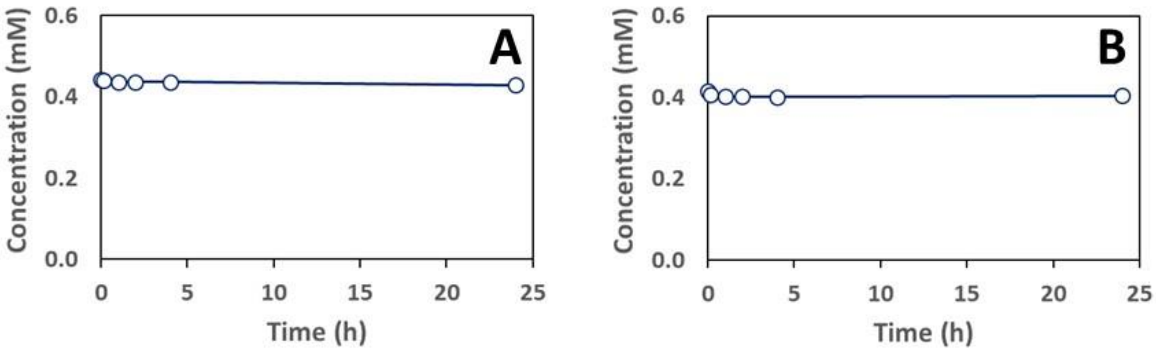

Figure S1. Vanillic acid concentration during in vitro enzyme assays of LigM (Saro_2861) (A) and DesA (Saro_2404) (B) without H_4folate. In both cases the substrate is not consumed after 24 hours.

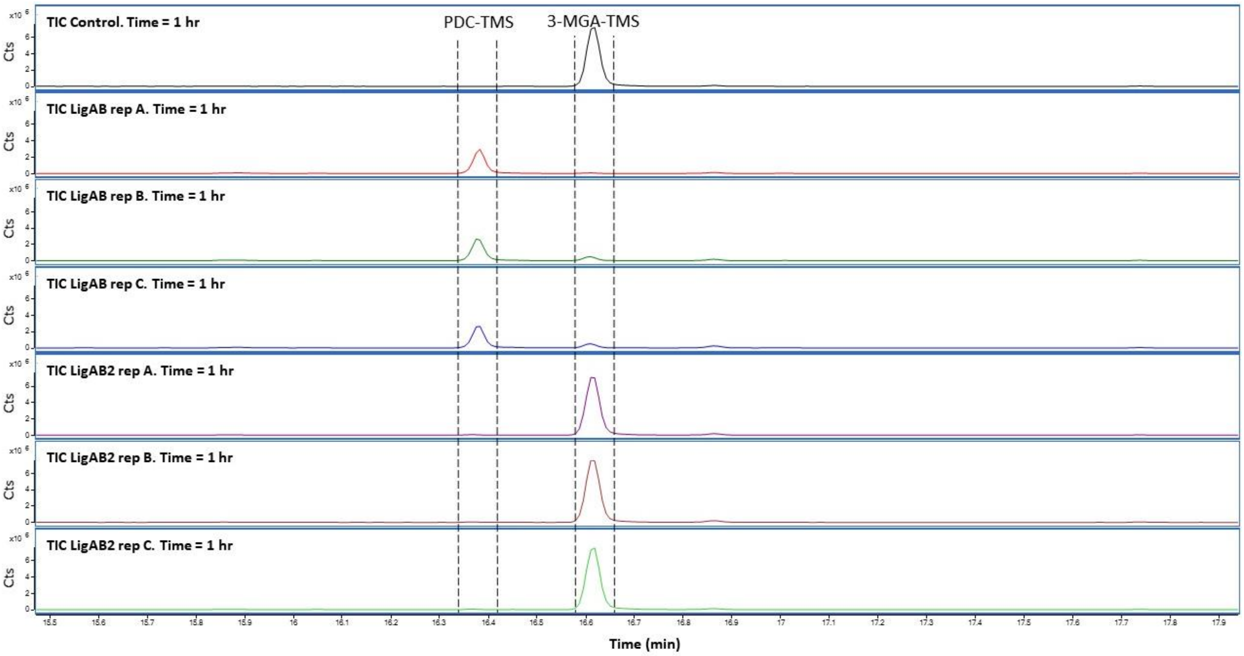

Figure S2. Total ion chromatogram of samples collected after 1 hour of reaction time of *in vitro* assays with LigAB and LigAB2 incubated in the presence of 3-MGA.

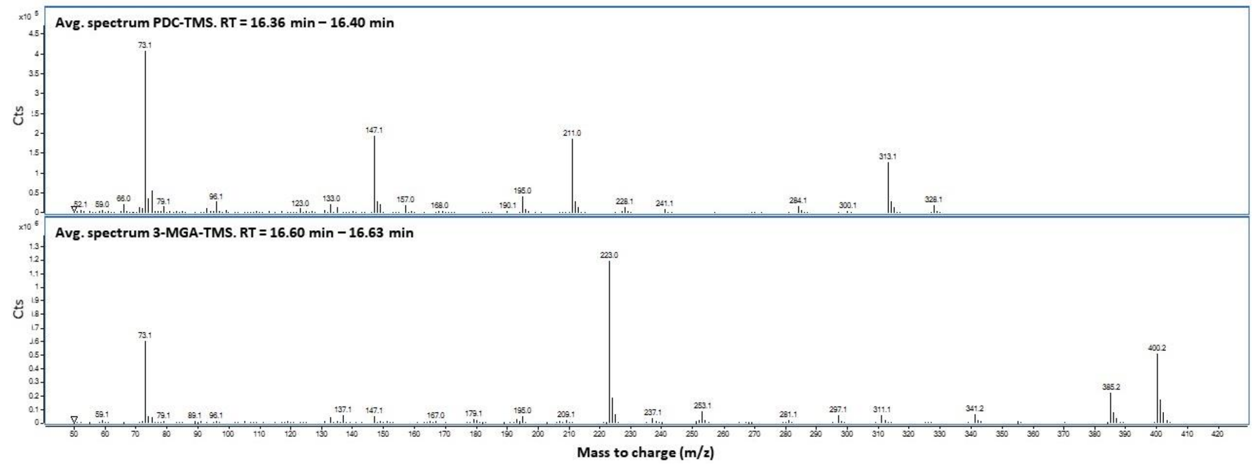

Figure S3. Average mass spectrum (m/z) of PDC (RT = 16.36 m – 16.40 m) and 3-MGA (RT = 16.60 m – 16.63 m) of samples collected after 1 hour of reaction time of *in vitro* assays with LigAB and LigAB2 incubated in the presence of 3-MGA.

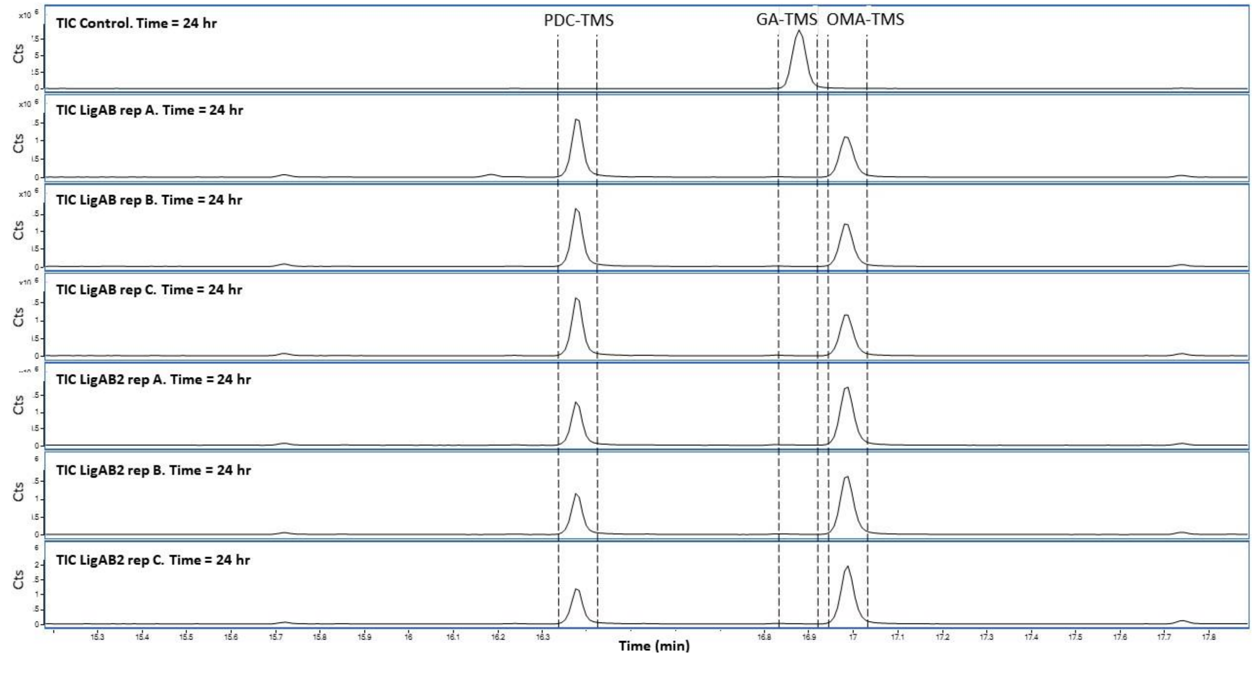

Figure S4. Total ion chromatogram of samples collected after 24 hour of reaction time of *in vitro* assays with LigAB and LigAB2 incubated in the presence of GA.

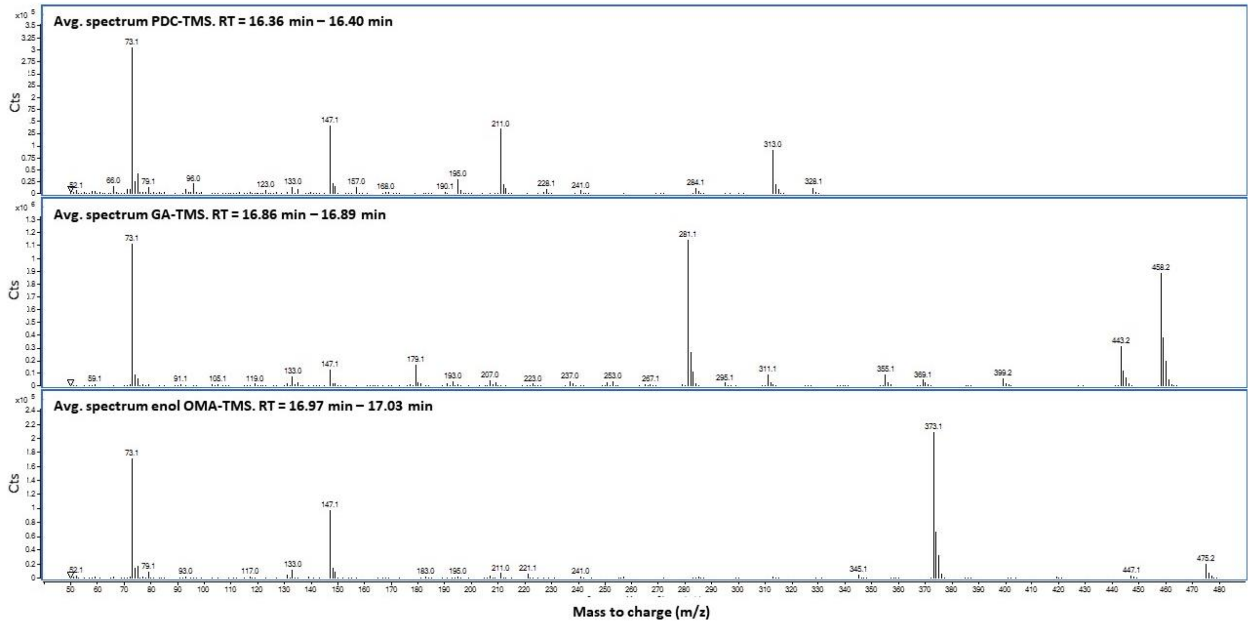

Figure S5. Average mass spectrum (m/z) of PDC-TMS (RT = 16.36m – 16.40 m), GA-TMS (RT = 16.86 m – 16.89 m), and enol OMA-TMS (RT = 16.97 m – 17.03 m) of samples collected after 24 hours of reaction time of *in vitro* assays with LigAB and LigAB2 incubated in the presence of GA.

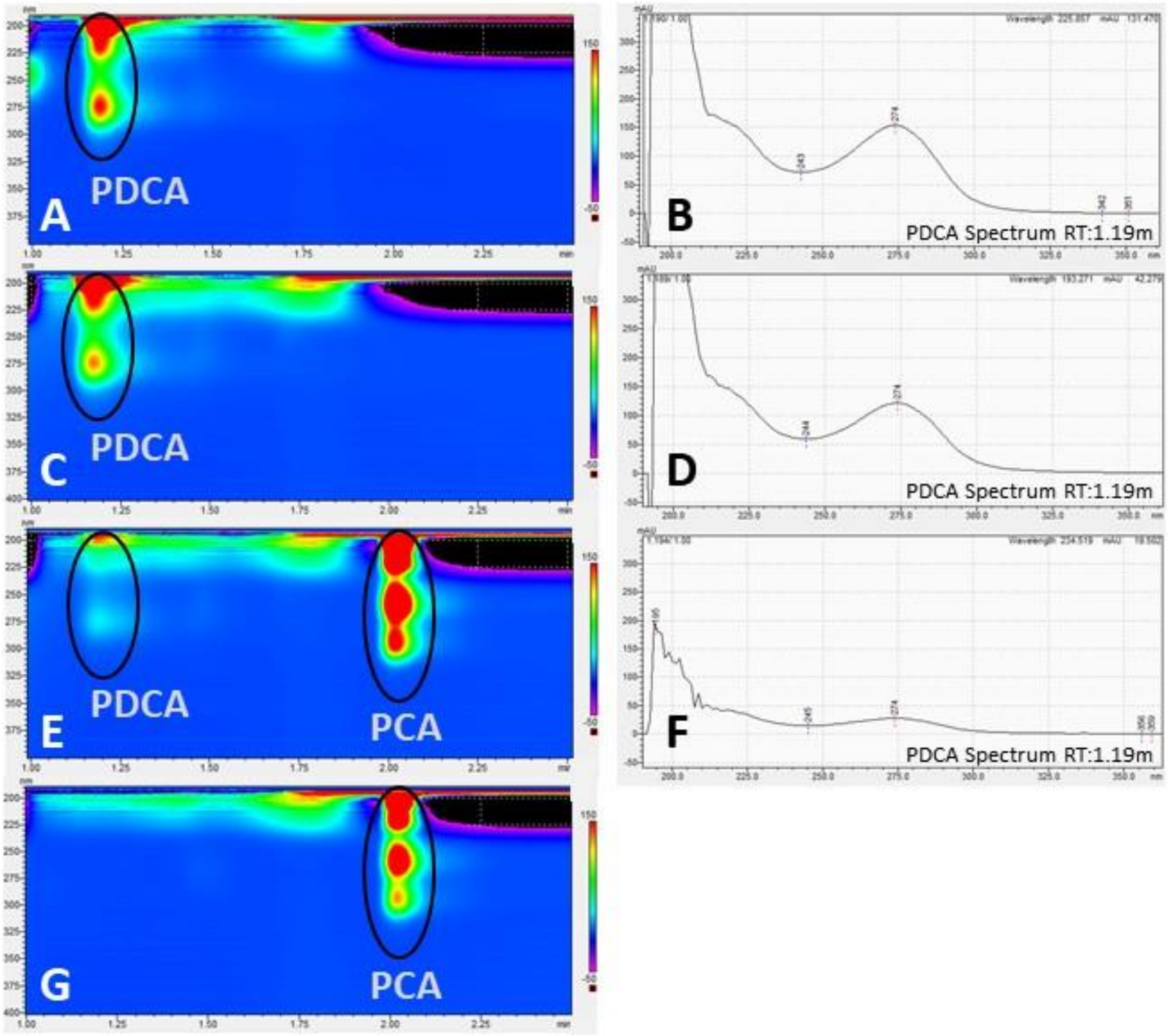

Figure S6. UV-vis chromatogram and spectra of authentic PDCA standard (A and B), ammonium sulfate treated samples collected at 24 hours from *in vitro* assays with LigAB and PCA (C and D), LigAB2 and PCA (E and F), and control without enzyme (G).

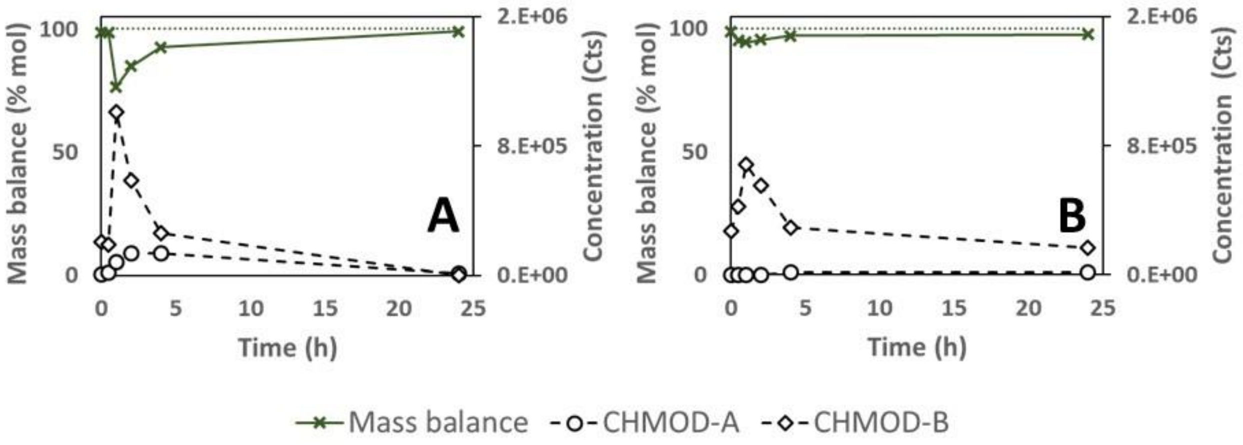

Figure S7. Mass balance and CHMOD relative concentration from *in vitro* assays of LigAB (Saro_2812/3) (A) and LigAB2 (Saro_1233/4) (B) with 3-MGA as a substrate. Mass balance was calculated as the sum of the molar concentrations of 3-MGA and PDC, divided by the concentration of 3-MGA in a sample lacking enzyme. Results are expressed in percentage (%). Dotted lines show 100%. Samples were analyzed with HPLC-MS, values correspond to the integrated area of peaks generated by the EIC with m/z = 215, attributed to CHMOD and concentrations correspond to the average of three replicates.

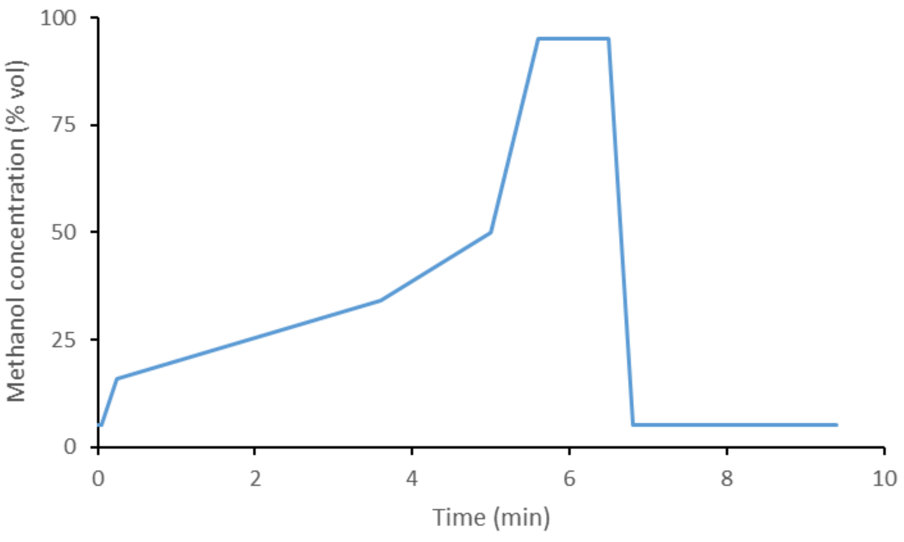

Figure S8. Binary gradient profile of HPLC-MS analysis

Table S1. Pairwise comparison of PDC yields of the indicated strains containing putative O-demethylases gene deletions with an unpaired, two-tailed t-test, with p ≤ 0.05 (bold) taken as significant.

**Strain 1**	**Strain 2**	**p-value Syringic Acid**	**p-value 3-MGA**	**p-value Vanillic Acid**
12444PDC	12444PDC*ΔligM*	**0.000318**	**0.000143**	0.164299
12444PDC	12444PDC*ΔdesA*	NA	0.793303	0.348259
12444PDC	12444PDC*ΔdmtS*	0.182088	0.138965	0.171743
12444PDC	12444PDC*ΔligMΔdesA*	**0.000016**	**0.013005**	**0.004528**
12444PDC	12444PDC*ΔligMΔdmtS*	**0.006359**	**0.003197**	0.392805
12444PDC	12444PDC*ΔdesAΔdmtS*	NA	**0.014495**	**0.039864**
12444PDC	12444PDC*ΔligMΔdesAΔdmtS*	NA	**0.001374**	**0.019908**
12444PDC*ΔligM*	12444PDC*ΔdesA*	NA	**0.000397**	0.083571
12444PDC*ΔligM*	12444PDC*ΔdmtS*	**0.005875**	**0.001037**	**0.047281**
12444PDC*ΔligM*	12444PDC*ΔligMΔdesA*	NA	0.287791	**0.003164**
12444PDC*ΔligM*	12444PDC*ΔligMΔdmtS*	**0.027134**	**0.017743**	0.094438
12444PDC*ΔligM*	12444PDC*ΔdesAΔdmtS*	NA	**0.001474**	**0.029413**
12444PDC*ΔligM*	12444PDC*ΔligMΔdesAΔdmtS*	NA	**0.006125**	**0.010263**
12444PDC*ΔdesA*	12444PDC*ΔdmtS*	NA	0.379338	0.945336
12444PDC*ΔdesA*	12444PDC*ΔligMΔdesA*	NA	**0.009770**	**0.001880**
12444PDC*ΔdesA*	12444PDC*ΔligMΔdmtS*	NA	**0.001707**	0.943976
12444PDC*ΔdesA*	12444PDC*ΔdesAΔdmtS*	NA	**0.008985**	0.517986
12444PDC*ΔdesA*	12444PDC*ΔligMΔdesAΔdmtS*	NA	**0.000551**	**0.006329**
12444PDC*ΔdmtS*	12444PDC*ΔligMΔdesA*	NA	**0.018943**	**0.003634**
12444PDC*ΔdmtS*	12444PDC*ΔligMΔdmtS*	**0.002793**	**0.005566**	0.880302
12444PDC*ΔdmtS*	12444PDC*ΔdesAΔdmtS*	NA	**0.015915**	0.394253
12444PDC*ΔdmtS*	12444PDC*ΔligMΔdesAΔdmtS*	NA	**0.003042**	**0.016204**
12444PDC*ΔligMΔdesA*	12444PDC*ΔligMΔdmtS*	NA	0.054664	**0.001826**
12444PDC*ΔligMΔdesA*	12444PDC*ΔdesAΔdmtS*	NA	**0.000859**	**0.004325**
12444PDC*ΔligMΔdesA*	12444PDC*ΔligMΔdesAΔdmtS*	NA	**0.023599**	0.083990
12444PDC*ΔligMΔdmtS*	12444PDC*ΔdesAΔdmtS*	NA	**0.000159**	0.476882
12444PDC*ΔligMΔdmtS*	12444PDC*ΔligMΔdesAΔdmtS*	NA	0.389459	**0.006005**
12444PDC*ΔdesAΔdmtS*	12444PDC*ΔligMΔdesAΔdmtS*	NA	**0.000079**	**0.021501**

Table S2. Multiple reaction module conditions for HPLC-MS quantification of compounds used in this study

Compound	MW (g/mol)	Parent (-) m/z	Transition 1	Transition 2	Transition 3
**PDC**	184.103	183.2	183.2 -> 111.1<br>CE13	183.2 -> 139.2<br>CE11	183.2 -> 95.1<br>CE11
**Gallic acid**	170.12	169.2	169.2 -> 125.1<br>CE16	169.2 -> 79.1<br>CE24	169.2 -> 97.1<br>CE21
**Protocatechuic acid**	154.12	153.1	153.1 -> 109.1<br>CE16	153.1 -> 108.1<br>CE24	153.1 -> 91.1<br>CE26
**3-MGA**	184.15	183.1	183.1 -> 168.2<br>CE16	183.1 -> 139.2<br>CE15	183.1 -> 123.1<br>CE27
**Vanillic acid**	168.15	167.1	167.1 -> 152.2<br>CE16	167.1 -> 108.1<br>CE17	167.1 -> 123.2<br>CE13
**Syringic acid**	198.17	197.1	197.1 -> 182.2<br>CE15	197.1 -> 95.1<br>CE29	197.1 -> 123.1<br>CE24

References

1. Kontur WS, Bingman CA, Olmsted CN, Wassarman DR, Ulbrich A, Gall DL, Smith RW, Yusko LM, Fox BG, Noguera DR, Coon JJ, Donohue TJ. 2018. Novosphingobium aromaticivorans uses a Nu-class glutathione S -transferase as a glutathione lyase in breaking the β-aryl ether bond of lignin. J Biol Chem 293:4955-4968.
2. Gall DL, Ralph J, Donohue T, Noguera D. 2014. A Group of Sequence-Related Sphingomonad Enzymes Catalyzes Cleavage of beta-Aryl Ether Linkages in Lignin beta-Guaiacyl and beta-Syringyl Ether Dimers. Environ Sci Technol 48:12454-12463.
3. Kontur WS, Olmsted CN, Yusko LM, Niles AV, Walters KA, Beebe ET, Vander Meulen KA, Karlen SD, Gall DL, Noguera DR, Donohue TJ. 2019. A heterodimeric glutathione S-transferase that stereospecifically breaks lignin's β(R)-aryl ether bond reveals the diversity of bacterial β-etherases. J Biol Chem 294:1877-1890.
4. Ono K, Nozaki M, Hayaishi O. 1970. Purification and some properties of protocatechuate 4,5-dioxygenase. Biochim Biophys Acta, Enzymol 220:224-238.
5. Kasai D, Masai E, Miyauchi K, Katayama Y, Fukuda M. 2015. Characterization of the gallate dioxygenase gene: Three distinct ring cleavage dioxygenases are involved in syringate degradation by Sphingomonas paucimobilis SYK-6. J Bacteriol 187:5067-5074.
6. Maruyama K. 1983. Purification and properties of 2-pyrone-4,6-dicarboxylate hydrolase. J Biochem 93:557-565.
